# Burden of tracheal, bronchus, and lung cancer in North Africa and Middle East countries, 1990 to 2019: Results from the GBD study 2019

**DOI:** 10.3389/fonc.2022.1098218

**Published:** 2023-02-10

**Authors:** Shaghayegh Khanmohammadi, Sahar Saeedi Moghaddam, Sina Azadnajafabad, Negar Rezaei, Zahra Esfahani, Nazila Rezaei, GBD 2019 NAME Tracheal, Bronchus and Lung Cancer Collaborators, Shaghayegh Khanmohammadi, Mohsen Naghavi, Bagher Larijani, Farshad Farzadfar

**Affiliations:** ^1^ Non-Communicable Diseases Research Center, Endocrinology and Metabolism Population Sciences Institute, Tehran University of Medical Sciences, Tehran, Iran; ^2^ Kiel Institute for the World Economy, Kiel, Germany; ^3^ Institute for Health Metrics and Evaluation, University of Washington, Seattle, WA, United States; ^4^ Department of Health Metrics Sciences, School of Medicine, University of Washington, Seattle, WA, United States; ^5^ Endocrinology and Metabolism Research Center, Endocrinology and Metabolism Clinical Sciences Institute, Tehran University of Medical Sciences, Tehran, Iran

**Keywords:** tracheal cancer, bronchus cancer, lung neoplasms, global burden of disease, attributable risks, tobacco use, incidence, death

## Abstract

**Objective:**

To provide estimates on the regional and national burden of tracheal, bronchus, and lung (TBL) cancer and its attributable risk factors from 1990 to 2019 in the North Africa and Middle East (NAME) region.

**Methods and materials:**

The Global Burden of Disease (GBD) 2019 data were used. Disability-adjusted life years (DALYs), death, incidence, and prevalence rates were categorized by sex and age groups in the NAME region, in 21 countries, from 1990 to 2019. Decomposition analysis was performed to calculate the proportion of responsible factors in the emergence of new cases. Data are presented as point estimates with their 95% uncertainty intervals (UIs).

**Results:**

In the NAME region, TBL cancer caused 15,396 and 57,114 deaths in women and men, respectively, in 2019. The age-standardized incidence rate (ASIR) increased by 0.7% (95% UI -20.6 to 24.1) and reached 16.8 per 100,000 (14.9 to 19.0) in 2019. All the age-standardized indices had a decreasing trend in men and an increasing trend in women from 1990 to 2019. Turkey (34.9 per 100,000 [27.6 to 43.5]) and Sudan (8.0 per 100,000 [5.2 to 12.5]) had the highest and lowest age-standardized prevalence rates (ASPRs) in 2019, respectively. The highest and lowest absolute slopes of change in ASPR, from 1990 to 2019, were seen in Bahrain (-50.0% (-63.6 to -31.7)) and the United Arab Emirates (-1.2% (-34.1 to 53.8)), respectively. The number of deaths attributable to risk factors was 58,816 (51,709 to 67,323) in 2019 and increased by 136.5%. Decomposition analysis showed that population growth and age structure change positively contributed to new incident cases. More than 80% of DALYs could be decreased by controlling risk factors, particularly tobacco use.

**Conclusion:**

The incidence, prevalence, and DALY rates of TBL cancer increased, and the death rate remained unchanged from 1990 to 2019. All the indices and contribution of risk factors decreased in men but increased in women. Tobacco is still the leading risk factor. Early diagnosis and tobacco cessation policies should be improved.

## 1 Introduction

Tracheal, bronchus, and lung (TBL) cancer is the second leading cause of new cancer cases and accounts for most of the cancer-related deaths worldwide (25% of all cancer deaths) (1). In 2019, a Global Burden of Disease (GBD) study reported 2.26 million new cases of TBL cancer and 2.04 million deaths and 45.9 million disability-adjusted life years (DALYs) due to TBL cancer (1). The chance of TBL cancer incidence is 2.6-fold higher in men than in women (2). The incidence of TBL cancer increases with age, and older people has a higher death rate due to TBL cancer (3).

Behavioral risk factors, such as smoking, diet, physical inactivity, air pollution, occupational exposure, and genetic factors, are among TBL cancer risk factors (4). Although the incidence of TBL cancer in high-sociodemographic-index (SDI) countries is higher than in low-SDI countries, the age-standardized incidence rate (ASIR) in high-SDI countries has been decreasing due to tobacco control in the last decade (2, 5). TBL cancer imposes a heavy economic burden to the countries globally (6–8). Non-specific and gradual clinical manifestations of TBL cancer have led to its late diagnosis and its poor prognosis, consequently. Although surgical techniques have improved in recent years, only a small portion of patients with TBL cancer undergo surgery with curative intent (9). Considering the aforementioned facts, appropriate policies could help to increase the survival rate of patients with TBL cancer (5).

As mentioned, the pattern of changes in incidence, deaths, and DALYs of TBL cancer is not the same worldwide and some regions, such as North Africa and Middle East (NAME), show an increasing trend, especially in women, in TBL burden (1). Thus, the NAME region calls for extra consideration. Improvements in the cancer registration system of the NAME region could be one of the reasons of this increasing trend. Poor tobacco control (the leading risk factor of TBL), increased number of women smokers, low level of awareness, and availability of data for recording the disease burden in NAME have led to poor TBL control in this region (10–14). Although TBL cancer is one of the preventable cancers worldwide, poor implementation of interventions for preventing TBL cancer in the NAME region has resulted in slow progress in cancer prevention since 2010. Despite improvements in the NAME region’s health indicator factors, there is still a significant socioeconomic disparity across and within the region’s countries, which has also affected the burden of TBL (15, 16). For instance, the ASIR of lung cancer in Tunisia is 15-fold higher in men than in Sudan, which could be due to the high number of unreported cases. Therefore, as one of the most common cancers, TBL cancer is one of the main concerns of the NAME region’s healthcare systems that needs to be addressed (10–13).

Comprehensive and up-to-date data regarding the TBL cancer burden may contribute to a better policymaking and management of this cancer in the NAME region due to the lack of sufficient evidence in this region. The GBD project reports an estimate of incidence, prevalence, mortality, years of life lost (YLLs), years lived with disability (YLDs), and DALYs of 369 diseases and injuries and their attributable risk factors (17, 18). Using findings of the GBD 2019, we report (a) estimates of the incidence, prevalence, deaths, YLLs, YLDs, and DALYs of TBL cancer and (b) estimates of attributable mortality, YLLs, YLDs, and DALYs for TBL cancer risk factors in the region, by country, sex, and age group between 1990 and 2019. Further, decomposition analysis was performed to calculate the proportion of responsible factors in the emergence of TBL new cases.

## 2 Materials and methods

Methodological details of GBD 2019 have previously been published (17, 18), where the burden of 369 diseases and injuries as well as attributed burden to 87 risk factors in 204 countries and territories by sex and age groups in terms of incidence, prevalence, death, DALYs, YLLs, and YLDs between 1990 and 2019 has been reported. Therefore, we have focused on the methods and statistical analyses of estimating TBL cancer.

### 2.1 Burden estimation framework

Data sources for 21 countries of the region (Afghanistan, Algeria, Bahrain, Egypt, Iran (Islamic Republic of), Iraq, Jordan, Kuwait, Lebanon, Libya, Morocco, Oman, Palestine, Qatar, Saudi Arabia, Sudan, Syrian Arab Republic, Tunisia, Turkey, United Arab Emirates, Yemen) were disease registries, surveys, reports, scientific literature, cancer registration, and vital registration for TBL cancer (**Supplementary Table 1**). Cause of Death Ensemble model (CODEm), spatiotemporal Gaussian process regression (ST-GPR), and DisMod-MR were the three main standardized tools to generate estimates by age, sex, location, and year (19). The burden of TBL cancer was calculated according to the International Classification of Diseases (ICD)-10 in GBD 2019. ICD-10 codes for mapping incidence were C33, C34-C34.92, Z12.2, Z80.1-Z80.2, and Z85.1-Z85.20 (20). In addition, ICD-10 codes for mapping deaths were C33-C34.9, D02.1-D02.3, D14.2-D14.3, and D38.1 (18).

### 2.2 Attributable burden estimation framework

The comparative risk assessment (CRA) conceptual framework was previously described by Murray and Lopez. CRA is the systematic evaluation of the changes in population health which result from changing the population distribution of exposure to a risk factor or a group of risk factors (21). GBD 2019 used this framework to calculate the burden of several causes and impairments attributable to 87 environmental and occupational, metabolic, and behavioral risks. Deaths, DALYs, YLLs, and YLDs attributable to risk factors were assessed. Smoking, secondhand smoke, residential radon, particulate matter pollution, occupational carcinogens, high fasting plasma glucose, and a diet low in fruits were identified as TBL cancer risk factors.

### 2.3 Statistical analysis

All the rates were reported as age-standardized (22) based on the GBD reference population, and numbers were expressed as all-ages. Age groups for TBL cancers started from 10 years. Uncertainty intervals (UIs) of 95% were calculated with the 2.5th and 97.5th percentiles of 1,000 drawn by age, sex, location, and year.

Decomposition analyses (23, 24) were conducted by calculating two scenarios to reveal the proportion of population growth, age structure changes, and incidence rate changes in the emergence of new cases between 1990 and 2019. First, we applied the age structure, sex structure, and age-specific rates from 1990 to the total population of the year 2019; then, we attributed the difference between the total number of cases in 1990 and the hypothetical scenario to population growth. In the second scenario, we applied the age-specific rates from 1990 to the age structure, sex structure, and population size of 2019. Differences between the second hypothetical scenario and the first hypothetical scenario were attributed to population aging and differences between the total number of cases in 2019 and the second hypothetical scenario were attributed to changes in the age-specific rates. Percent changes were calculated as the change between burden in 2019 and 1990 divided by the burden in 1990. Figures and tables were illustrated by R version 3.4.2.

To demonstrate the time difference by country, we divided the highest rate (age-standardized) for each index among countries to the lowest rate in 1990 and 2019. For demonstrating the time difference by sex, we divided the rate of each index for men to women in 1990 and 2019.

## 3 Results

### 3.1 TBL cancer incidence, prevalence, mortality, YLLs, YLDs, and DALYs

**Figure 1:**
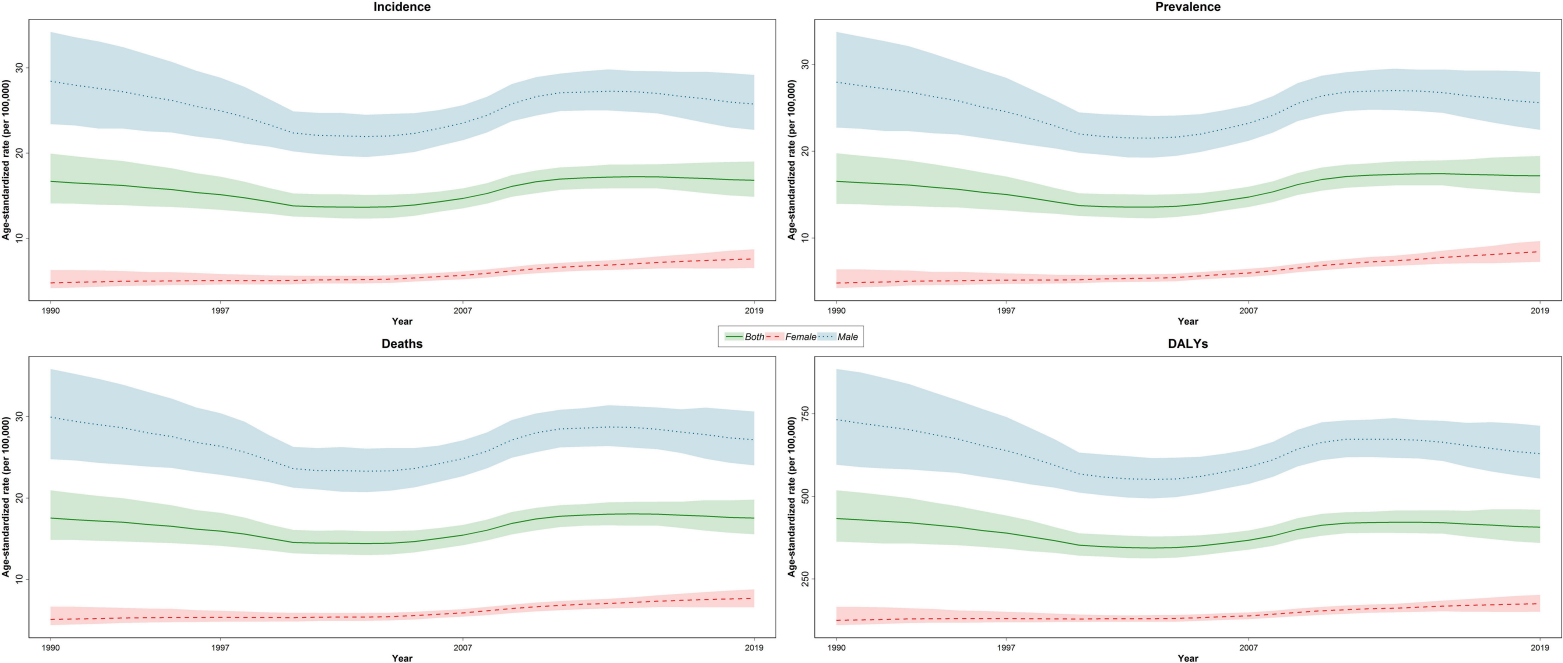
Time trend of age-standardized rate of incidence, prevalence, deaths, and DALYs by sex in region *DALYs* = *disability-adjusted life years*.

**Figure 2:**
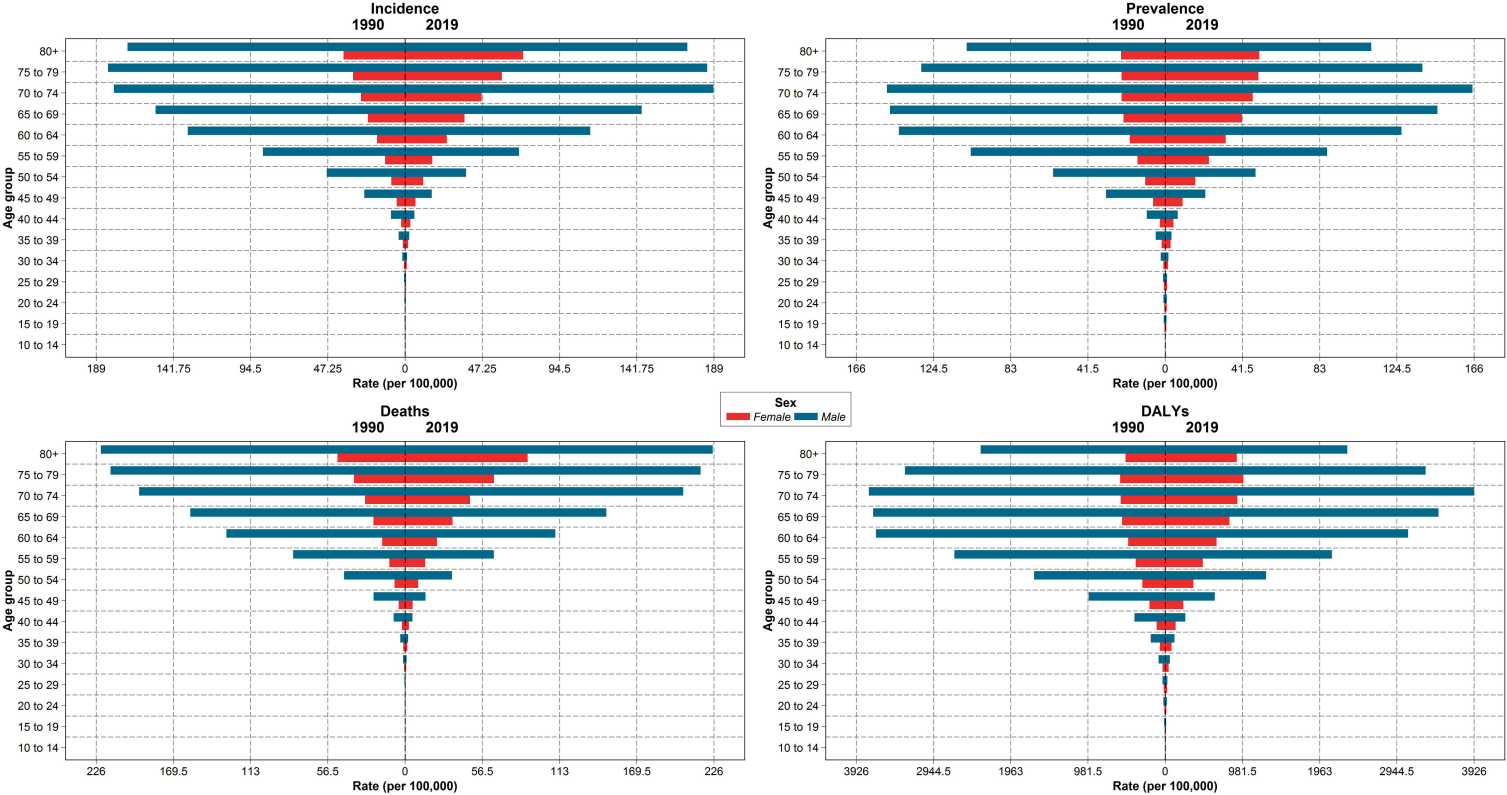
The incidence, prevalence, deaths, and DALY rates by age groups and sex in region, 1990 compared with 2019 *DALYs = disability-adjusted life years*.

The new cases of TBL cancer were 29,046 (24,454 to 34,695) in 1990. The number of all-age incident cases of TBL cancer in 2019 was 71,681 (63,424 to 81,049) and changed by 146.8% between 1990 and 2019 in the NAME region. The number of deaths was 72,510 (64,113 to 81,925) in 2019 and changed by 145.4% between 1990 and 2019. The ASIR of TBL cancer increased by 0.7% (95% UI -20.6 to 24.1) from 16.7 per 100,000 (14.1 to 19.9) in 1990 to 16.8/100,000 persons (14.9 to 19.0) in 2019 in the region. The change in ASIR between 1990 and 2019 was 58.1% (9.6 to 96.7) and -9.4% (-28.8 to 15.6) in women and men, respectively. The age-standardized prevalence rate (ASPR) of TBL cancer showed a slight increase and was 16.5 per 100,000 (14.0 to 19.8) in 1990 and 17.2 per 100,000 (15.2 to 19.5) in 2019. Similar to ASIR, ASPR decreased (-8.5% [-28.5 to 17.1]) between 1990 and 2019 in men but rose in women (75.1% [20.3 to 115.7]). The age-standardized death rate (ASDR) was similar in 1990 and 2019 (1990: 17.5 [14.8 to 21.0], 2019: 17.5 [15.6 to 19.8]). Although age-standardized DALYs and age-standardized YLL rates showed a downward trend between 1990 and 2019, there was a small increase in age-standardized YLDs. TBL cancer accounted for 433.2 (363.4 to 518.3) age-standardized DALYs in 1990 and 406.7 (359.1 to 459.5) DALYs in 2019 (**Table 1**). There has been a decrease in time difference of ASIR (5.9 vs. 3.4), ASPR (5.8 vs. 3.0), ASDR (5.9 vs. 3.5), DALYs (5.9 vs. 3.6), YLLs (5.9 vs. 3.6), and YLDs (5.3 vs. 3.2) rate by sex between 1990 and 2019. It is noticeable that all the indices had an increasing trend in women, but they decreased in men (**Figure 1**). In 1990 and 2019, the incidence rate, rates of prevalence, DALYs, and deaths due to TBL cancer in both sexes increased with age. However, the rates of TBL cancer DALYs, prevalence, and incidence in men began to decrease from 74 and 80 and the rate of DALYs decreased in women over 80 years old (**Figure 2**).

**Table 1:** Age-standardized rate (per 100,000) of incidence, prevalence, deaths, DALYs, YLLs, and YLDs in region by sex in 1990 and 2019 with their percent changes.

Measure	Sex	Age-standardized rate (per 100,000)		% Change (1990 to 2019)
		1990	2019	
Incidence	Both	16.7 (14.1 to 19.9)	16.8 (14.9 to 19.0)	0.7 (-20.6 to 24.1)
	Female	4.8 (4.2 to 6.3)	7.6 (6.6 to 8.7)	58.1 (9.6 to 96.7)
	Male	28.4 (23.4 to 34.2)	25.8 (22.7 to 29.2)	-9.4 (-28.8 to 15.6)
Prevalence	Both	16.5 (14.0 to 19.8)	17.2 (15.2 to 19.5)	3.8 (-18.7 to 28.7)
	Female	4.8 (4.2 to 6.4)	8.4 (7.3 to 9.7)	75.1 (20.3 to 115.7)
	Male	28.0 (22.7 to 33.7)	25.6 (22.5 to 29.1)	-8.5 (-28.5 to 17.1)
Deaths	Both	17.5 (14.8 to 21.0)	17.5 (15.6 to 19.8)	0.0 (-21.1 to 22.4)
	Female	5.1 (4.4 to 6.7)	7.7 (6.6 to 8.8)	51.2 (4.6 to 89.5)
	Male	30.0 (24.8 to 35.9)	27.2 (24.0 to 30.6)	-9.3 (-28.5 to 14.5)
DALYs	Both	433.2 (363.4 to 518.3)	406.7 (359.1 to 459.5)	-6.1 (-26.2 to 16.9)
	Female	124.7 (110.3 to 165.4)	174.9 (151.1 to 201.6)	40.3 (-4.9 to 73.4)
	Male	732.4 (596 to 885.6)	629.3 (554.1 to 713.7)	-14.1 (-32.9 to 10.2)
YLLs	Both	429.5 (360.0 to 513.7)	402.9 (355.4 to 455.9)	-6.2 (-26.3 to 16.8)
	Female	123.5 (109.3 to 163.8)	173.1 (149.5 to 199.5)	40.2 (-5.0 to 73.2)
	Male	726.1 (590.6 to 877.9)	623.5 (548.6 to 707.3)	-14.1 (-32.9 to 10.2)
YLDs	Both	3.8 (2.6 to 5.1)	3.8 (2.7 to 5.0	0.4 (-21.7 to 25.2)
	Female	1.2 (0.8 to 1.6)	1.8 (1.2 to 2.4)	55.4 (5.4 to 99.6)
	Male	6.4 (4.4 to 8.8)	5.7 (4.1 to 7.6)	-9.8 (-30.8 to 16.6)

Data in parentheses are 95% uncertainty interval (95% UI).

DALYs, disability-adjusted life years; YLLs, years of life lost; YLDs, years lived with disability.

The decomposition analysis revealed that the incident cases grew by 146.8% (93.4 to 206.6) between 1990 and 2019; of these cases, 76.4%, 77.3%, and -7.0% were attributable to population growth, age structure change, and the disease-incidence rate change, respectively (**Table 2**). In 1990, there were 4,120 (3,627 to 5,485) incident cases in women that increased by 284.4% (73.8%, 78.4%, and 132.2% attributed to population growth, age structure change, and incidence rate change, respectively) to 15,838 (13,642 to 18,196) in 2019. In comparison with women, incident cases in men increased by 124.0% (75.0 to 186.8) between 1990 and 2019, of which 78.9%, 76.9%, and -31.8% were attributable to population growth, age structure change, and incidence rate change, respectively (**Supplementary Table 2**).

**Table 2:** Decomposition analysis of new cases change cause between 1990 and 2019.

Location		New cases		Expected new cases in 2019		% 1990–2019 new cases change cause			% 1990–2019 new cases overall change
		1990	2019	Population growth	Population growth + aging	Population growth	Age structure change	Incidence rate change	
**North Africa and Middle East**		29,046	71,681	51,244	73,701	76.4%	77.3%	-7.0%	146.8%
**Country**	Afghanistan	948	1,476	3,177	1,612	235.2%	-165.2%	-14.3%	55.7%
	Algeria	1,375	3,193	2,275	3,857	65.5%	115.1%	-48.3%	132.3%
	Bahrain	59	141	169	336	184.0%	281.3%	-328.8%	136.5%
	Egypt	2,018	6,123	3,589	4,452	77.9%	42.8%	82.8%	203.5%
	Iran (Islamic Republic of)	2,865	8,705	4,126	8,001	44.0%	135.2%	24.6%	203.8%
	Iraq	1,219	4,154	2,917	3,654	139.4%	60.4%	41.1%	240.9%
	Jordan	167	914	516	836	208.4%	191.8%	46.8%	447.0%
	Kuwait	69	225	174	303	151.6%	185.2%	-111.6%	225.3%
	Lebanon	482	1,421	762	1,085	58.1%	66.9%	69.8%	194.8%
	Libya	365	925	581	1,001	59.0%	114.9%	-20.8%	153.1%
	Morocco	2,055	5,277	2,921	4,781	42.1%	90.5%	24.2%	156.8%
	Oman	59	147	139	162	135.9%	38.1%	-25.4%	148.6%
	Palestine	173	523	415	473	139.4%	33.7%	29.2%	202.3%
	Qatar	17	125	107	158	543.5%	300.2%	-196.3%	647.4%
	Saudi Arabia	422	1,545	939	1,292	122.7%	83.7%	60.1%	266.4%
	Sudan	715	1,525	1,445	1,427	102.0%	-2.5%	13.6%	113.1%
	Syrian Arab Republic	544	1,372	611	1,280	12.4%	123.1%	16.9%	152.3%
	Tunisia	962	2,462	1,319	2,437	37.1%	116.2%	2.6%	155.9%
	Turkey	13,983	29,511	19,033	34,271	36.1%	109.0%	-34.0%	111.0%
	United Arab Emirates	62	542	306	569	393.7%	423.6%	-44.7%	772.5%
	Yemen	467	1,302	1,071	1,264	129.5%	41.4%	8.2%	179.0%

**Figure 3:**
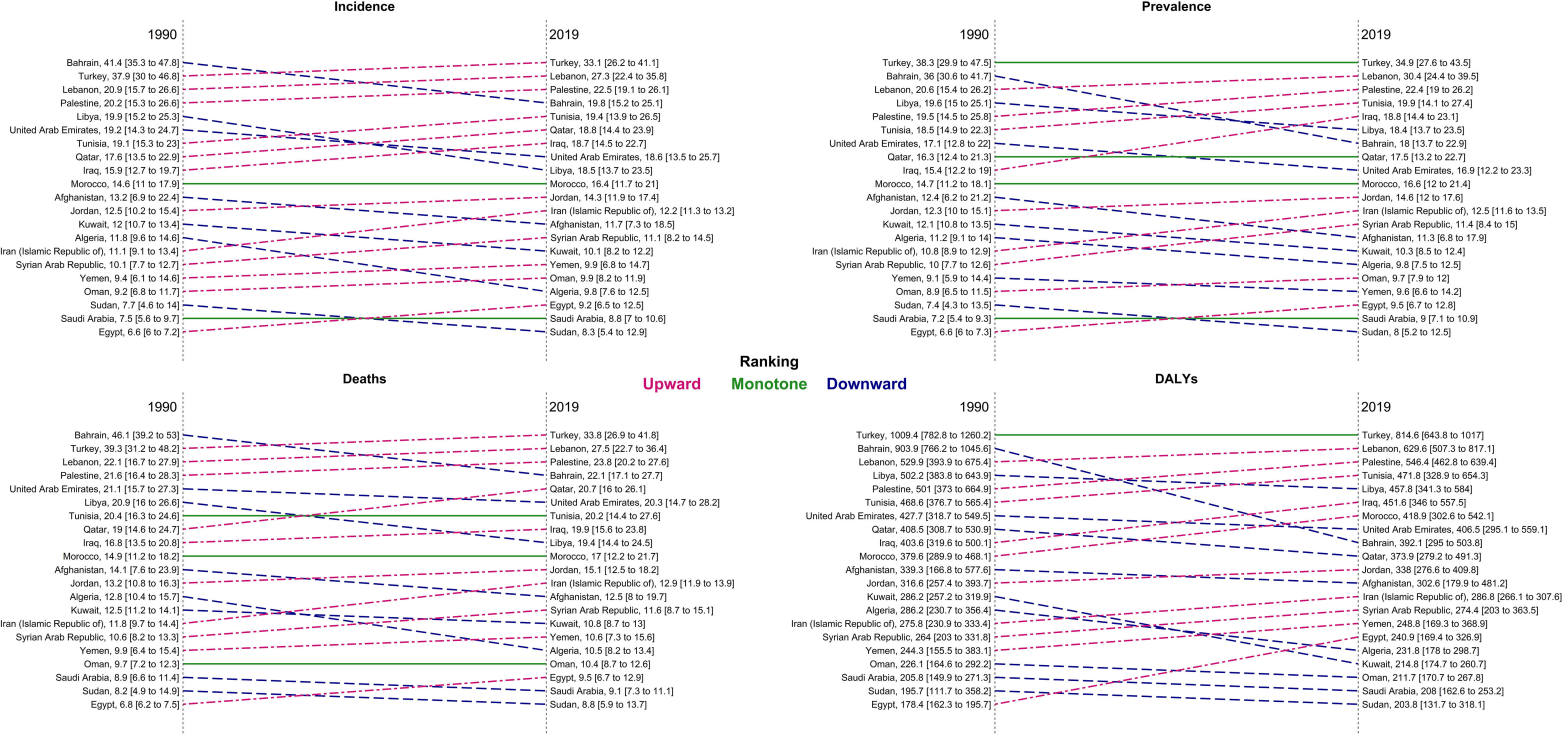
Ranking of age-standardized rate of incidence, prevalence, deaths, and DALYs in countries of the region, 1990 compared with 2019 *DALYs = disability-adjusted life years*.

At the national level, in 1990, Bahrain, Turkey, and Lebanon had the highest ASIR, ASDR, ASPR, and age-standardized DALYs rate, but in 2019, the respective measures were the highest in Turkey, Lebanon, and Palestine (**Figure 3**). Bahrain had the highest ASIR (41.4 [35.3 to 47.8]) and ASDR (46.1 [39.2 to 53.1]) in 1990, which decreased to 19.8 (15.2 to 25.1) and 22.1 (17.1 to 27.7) in 2019, respectively. Turkey had the highest rate of ASPR (1990: 38.3 [29.9 to 47.5], 2019: 34.9 persons [27.6 to 43.5]) and DALYs (1990: 1009.4 [782.8 to 1260.2], 2019: 814.6 [643.8 to 1017.0]) between 1990 and 2019, with -8.7% (-35.4 to 26.9) and -19.3% (-43.1 to 12.7) change in ASPR and age-standardized DALY rates, respectively. Egypt had the lowest ASIR, ASPR, ASDR, and age-standardized DALYs rate in 1990; however, all the indices were increased by 40.2% (-4.5 to 92.4), 42.9% (-3.5 to 96.3), 39.3% (-4.9 to 91.5), and 35.0% (-8.4 to 85.7) in 2019, respectively. Overall, Afghanistan, Algeria, Bahrain, Kuwait, Libya, Turkey, and United Arab Emirates had a decreasing trend in all the indices between 1990 and 2019, but all the indices of Egypt, Lebanon, Morocco, and Tunisia grew between 1990 and 2019 in both men and women. There has been a decrease in the time difference of ASIR (6.3 vs. 4.0), ASPR (5.8 vs. 4.4), ASDR (6.8 vs. 3.8), DALY rate (5.7 vs. 4.0), YLD rate (5.5 vs. 3.9), and YLL rate (5.7 vs. 4.0) by country, between 1990 and 2019. In most of the countries of the region, an upward trend in the incidence, prevalence, death, DALY, YLL, and YLD rates of age-standardized TBL cancer in women between 1990 and 2019 was observed (**Supplementary Table 3**).

In all the countries of the region, the number of new cases was higher in 2019 compared with 1990. Our decomposition analysis showed that, except for Sudan and Afghanistan, age structure changes positively contributed to the increase of new incident cases. Bahrain had the highest negative new cases change cause, attributed to the incident rate change (-328.8%) (**Table 2**).

### 3.2 TBL cancer burden attributable to risk factors

**Figure 4:**
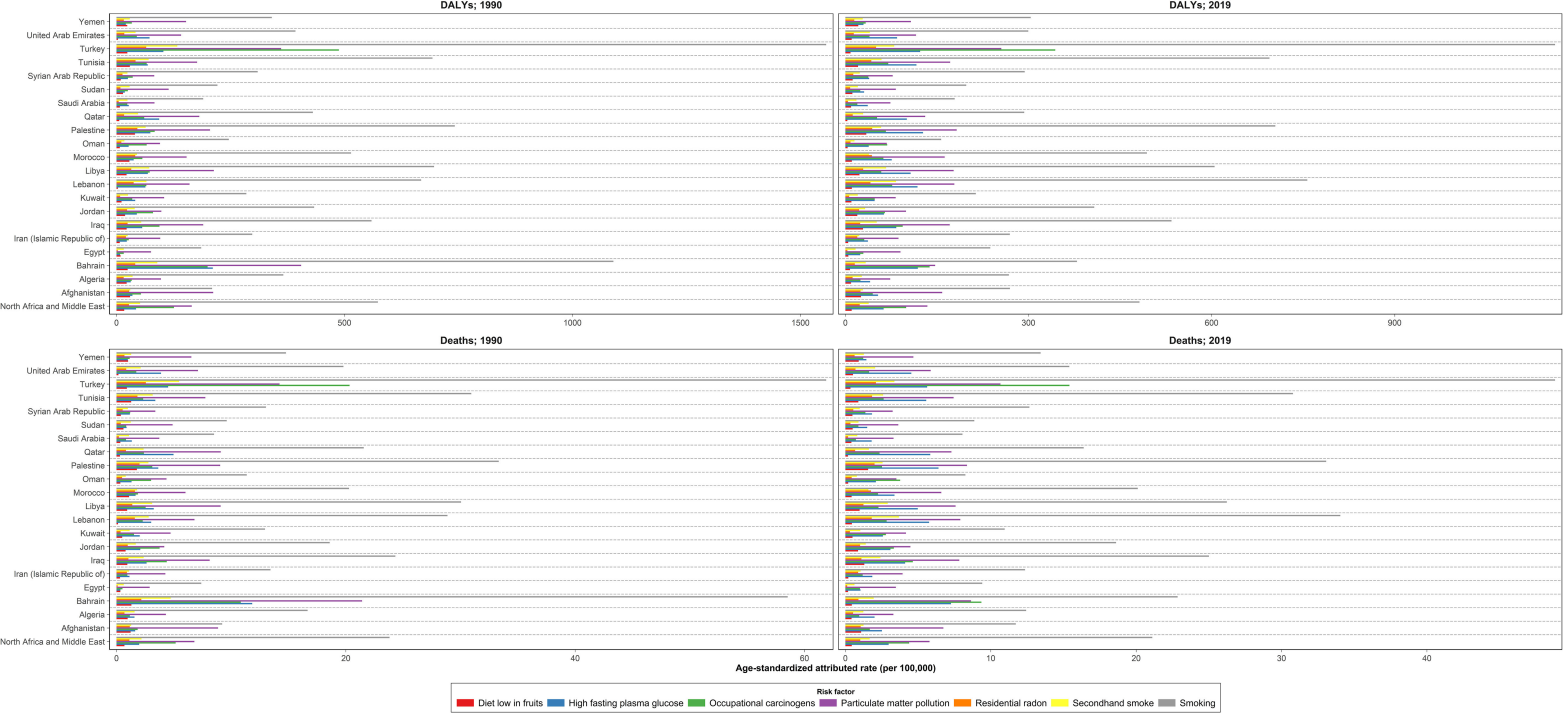
Age-standardized DALYs and death rates due to tracheal, bronchus, and lung cancer attributed to risk factors in the region and countries, 1990 and 2019 DALYs = disability-adjusted life years.

The number of deaths attributable to risk factors was 58,816 (51,709.2 to 67,322.9) in 2019 and increased by 136.5% between 1990 and 2019 in the NAME region. The DALYs attributable to risk factors were 1,488,965.7 (1,301,197.0 to 1,708,995.5) in 2019 and changed by 122.7% between 1990 and 2019. Overall, the TBL cancer ASDR, age-standardized DALY rate, YLL rate, and YLD rate attributable to all risk factors decreased between 1990 and 2019. TBL cancer ASDR, DALY rate, YLL rate, and YLD rate attributable to all risk factors decreased in men by -10.3% (-29.6 to 13.9), -15.0% (-33.9 to 9.3), -15.1% (-33.9 to 9.3), and -10.7% (-31.5 to 15.8) between 1990 and 2019, respectively. Unlike men, TBL cancer ASDR, DALY rate, YLL rate, and YLD rate attributable to all risk factors rose in women by 49.4% (3.5 to 90.8), 38.3% (-5.8 to 74.9), 38.2% (-5.9 to 74.9), and 54.0% (3.8 to 101.0) between 1990 and 2019, respectively. In all the countries of the region, except for Kuwait and Bahrain, indices attributable to all risk factors increased in women between 1990 and 2019. The YLD attributable risk factors reflected the same pattern of attributable deaths (**Table 3** and **Supplementary Table 4**). Tobacco use was the leading cause of deaths and DALY rate of TBL cancer in both sexes and all age groups (except DALY rate under 40). The attributed death rate to tobacco decreased by -7.2% from 1990 to 2019. After tobacco, air pollution, occupational risks, high fasting plasma glucose, residential radon, and a diet low in fruits had the highest attributed DALY rate in 1990 and 2019, respectively. Behavioral, environmental/occupational, and metabolic risks related to DALY and death rates had increased in women between 1990 and 2019. Unlike women, only the DALY and death rates attributable to high fasting plasma glucose grew in men between 1990 and 2019. Considering both sexes, except for the TBL cancer death rate attributable to high fasting plasma glucose (66.3% [34.2 to 113.3]) and residential radon (0.5% [-21.3 to 24.7]), the attributed death rate to other risk factors decreased between 1990 and 2019. Bahrain, followed by Turkey, had the highest death rate attributable to all risk factors and their subgroups in 1990; however, the highest death rate attributable to all risk factors and their subgroups in Bahrain dropped drastically between 1990 and 2019. Despite the current trend in Bahrain, the highest death rate attributable to all risk factors was observed in Turkey, Lebanon, and Bahrain (**Figure 4**; **Supplementary Figure** and **Table 4**).

**Table 3:** Attributed age-standardized rate (per 100,000) of deaths, DALYs, YLLs, and YLDs to all risk factors in region by sex in 1990 and 2019 with their percent changes.

Measure	Sex	Attributed age-standardized rate (per 100,000)		% Change (1990 to 2019)
		1990	2019	
Deaths	Both	14.8 (12.4 to 17.8)	14.3 (12.6 to 16.3)	-3.7 (-24.2 to 19.7)
	Female	2.9 (2.4 to 3.9)	4.4 (3.6 to 5.3)	49.4 (3.5 to 90.8)
	Male	26.7 (22.1 to 32.0)	24.0 (21.0 to 27.2)	-10.3 (-29.6 to 13.9)
DALYs	Both	362.8 (299.1 to 436.1)	328.0 (287.7 to 375.7)	-9.6 (-29.6 to 13.6)
	Female	70.8 (59.0 to 94.8)	98.0 (80.4 to 118.7)	38.3 (-5.8 to 74.9)
	Male	646.2 (526.5 to 781.7)	549.1 (480.2 to 628.4)	-15.0 (-33.9 to 9.3)
YLLs	Both	359.7 (297.0 to 432.4)	325.0 (285.0 to 372.0)	-9.6 (-29.6 to 13.5)
	Female	70.2 (58.5 to 94.1)	96.9 (79.6 to 117.6)	38.2 (-5.9 to 74.9)
	Male	640.6 (520.8 to 774.4)	544.1 (476.0 to 622.1)	-15.1 (-33.9 to 9.3)
YLDs	Both	3.2 (2.2 to 4.3)	3.1 (2.2 to 4.1)	-3.6 (-25.4 to 21.5)
	Female	0.7 (0.4 to 1.0)	1.0 (0.7 to 1.4)	54.0 (3.8 to 101)
	Male	5.6 (3.9 to 7.8)	5.0 (3.6 to 6.7)	-10.7 (-31.5 to 15.8)

Data in parentheses are 95% uncertainty interval (95% UI).

DALYs, disability-adjusted life years; YLLs, years of life lost; YLDs, years lived with disability.

**Table 4:** Attributed age-standardized rate (per 100,000) of DALYs and deaths by risk factors in region by sex in 1990 and 2019 with their percent changes.

Risk factor	Measure	Sex	Attributed age-standardized rate (per 100,000)		% Change (1990 to 2019)
			1990	2019	
Smoking	Deaths	Both	12.6 (10.5 to 15.0)	11.6 (10.2 to 13.2)	-7.5 (-27.1 to 16.3)
		Female	1.3 (1 to 1.7)	1.9 (1.6 to 2.2)	46.6 (3.2 to 93.4)
		Male	23.8 (19.9 to 28.5)	21.1 (18.6 to 24.0)	-11.3 (-30.2 to 12.6)
	DALYs	Both	306.1 (251.4 to 369.1)	266 (232.1 to 304.2)	-13.1 (-32.3 to 10.8)
		Female	30.9 (25.2 to 41.7)	41.5 (34.6 to 49.1)	34.3 (-7 to 74.3)
		Male	573.2 (468 to 692.9)	481.8 (420.6 to 552.1)	-15.9 (-34.6 to 7.8)
Particulate matter pollution	Deaths	Both	4.0 (2.8 to 5.4)	3.8 (2.8 to 4.8)	-5.9 (-27.2 to 16.8)
		Female	1.2 (0.9 to 1.7)	1.7 (1.2 to 2.2)	40.7 (-3.4 to 79.8)
		Male	6.8 (4.8 to 9.2)	5.8 (4.3 to 7.5)	-14.8 (-34.4 to 8.3)
	DALYs	Both	98.3 (69.8 to 132.3)	87.7 (64.5 to 112.7)	-10.8 (-31.4 to 11.2)
		Female	29.5 (21.6 to 40.9)	39.1 (28.4 to 50.6)	32.4 (-10.3 to 66.5)
		Male	165 (115.5 to 224.1)	134.3 (99 to 173.9)	-18.6 (-37.8 to 4.4)
Occupational carcinogens	Deaths	Both	2.7 (1.9 to 3.7)	2.4 (1.7 to 3.4)	-11.8 (-37.6 to 24.2)
		Female	0.3 (0.2 to 0.4)	0.4 (0.2 to 0.5)	35.7 (-18.5 to 119.7)
		Male	5.2 (3.5 to 7.1)	4.4 (3.0 to 6.2)	-14.9 (-40.8 to 21.3)
	DALYs	Both	66.9 (44.8 to 92.9)	54.3 (37.4 to 75.7)	-18.8 (-42.7 to 15.2)
		Female	6.1 (3.7 to 9.3)	7.3 (4.8 to 10.7)	20.7 (-27.0 to 88.4)
		Male	125.9 (83.4 to 175.2)	99.5 (67.6 to 140.5)	-20.9 (-44.7 to 13.1)
Secondhand smoke	Deaths	Both	1.4 (0.8 to 2.1)	1.2 (0.7 to 1.8)	-14.2 (-31.9 to 5.5)
		Female	0.5 (0.3 to 0.8)	0.7 (0.4 to 1.0)	21.7 (-16.9 to 52.1)
		Male	2.2 (1.2 to 3.4)	1.7 (1.0 to 2.5)	-23.5 (-39.8 to -2.0)
	DALYs	Both	32.9 (19.2 to 50.2)	27.2 (16.4 to 41.6)	-17.2 (-34.5 to 2.4)
		Female	13.8 (8.3 to 20.6)	16 (9.5 to 24.0)	16.6 (-22.0 to 43.7)
		Male	51.5 (29.5 to 80.6)	38 (22.8 to 58.9)	-26.1 (-43.1 to -3.9)
High fasting plasma glucose	Deaths	Both	1.1 (0.2 to 2.7)	1.9 (0.4 to 4.2)	66.3 (34.2 to 113.3)
		Female	0.3 (0.1 to 0.8)	0.8 (0.2 to 1.9)	151.8 (75.5 to 231.2)
		Male	2.0 (0.3 to 4.9)	3.0 (0.5 to 6.9)	50.6 (21.5 to 95.3)
	DALYs	Both	25.0 (4.6 to 60.8)	39.9 (8.6 to 88.6)	59.6 (26.8 to 106.3)
		Female	6.9 (1.3 to 16.4)	16.5 (3.4 to 39.4)	140.4 (66.4 to 210.7)
		Male	42.8 (6.6 to 106.7)	62.6 (10.4 to 144.7)	46.4 (16.6 to 93.0)
Residential radon	Deaths	Both	0.7 (0.1 to 1.4)	0.7 (0.1 to 1.3)	0.5 (-21.3 to 24.7)
		Female	0.2 (0.0 to 0.4)	0.3 (0.0 to 0.6)	56.6 (7.5 to 97.9)
		Male	1.1 (0.2 to 2.4)	1.0 (0.2 to 2.1)	-9.0 (-29.4 to 14.6)
	DALYs	Both	16.0 (2.7 to 34.1)	15 (2.5 to 30.5)	-6.2 (-27.5 to 17.3)
		Female	4.4 (0.7 to 9.0)	6.2 (1.0 to 12.8)	43.3 (-2.7 to 79.5)
		Male	27.3 (4.7 to 59.0)	23.4 (3.8 to 47.5)	-14.2 (-34.1 to 10.6)
Diet low in fruits	Deaths	Both	0.4 (0.1 to 0.7)	0.3 (0.1 to 0.4)	-28.9 (-42.9 to -10.3)
		Female	0.1 (0.0 to 0.2)	0.1 (0.0 to 0.2)	9.7 (-24.7 to 43.4)
		Male	0.7 (0.2 to 1.1)	0.4 (0.1 to 0.7)	-37.0 (-48.7 to -17.8)
	DALYs	Both	10.2 (2.7 to 16.4)	7.0 (2.1 to 10.6)	-31.6 (-45.0 to -12.8)
		Female	3.4 (0.9 to 5.4)	3.6 (1.1 to 5.5)	6.5 (-27.0 to 37.1)
		Male	16.9 (4.5 to 27.6)	10.3 (3.1 to 15.5)	-39.3 (-51.1 to -20.0)

Data in parentheses are 95% uncertainty interval (95% UI); DALYs, disability-adjusted life years.

## 4 Discussion

In this study, we analyzed the results of the GBD 2019 study to determine the regional and national burden of TBL cancer and its attributable risk factors in the North Africa and Middle East region. Our analysis showed that the regional ASIR of TBL cancer slightly increased and the ASDR remained unchanged, but the age-standardized DALY rate decreased between 1990 and 2019. All the indices had a marked increase in women but decreased in men.

Studies on the global and regional trends of TBL cancer from the GLOBOCAN database also revealed an increasing and decreasing trend in TBL cancer incidence in women and men, respectively (25, 26). Smoking as the main risk factor of TBL cancer is steadily increasing in women of the NAME region, which is contrary to men (27, 28). Previously, tobacco was considered taboo among women; however, due to social media, globalization, and marketing efforts, it is changing into a “normal” behavior among women in the NAME region, which is a public health concern (27). In addition, several studies have suggested that smoking has more harmful effects on women than men (29). New types of smoking, such as water pipes, are also increasing in the NAME region (30). Not only smoking but also opium, which is prevalently used in the NAME region, increases the risk lung cancer (31). Moreover, late diagnosis of TBL cancer leads to a poor prognosis in patients (32); the government and health system of the NAME region do not conduct appropriate screening tests and have failed to establish proper rules regarding primary prevention of TBL cancer (10), which may have resulted in an unchanged death rate and an increased incidence rate of TBL cancer between 1990 and 2019.

Bahrain, Turkey, and Lebanon had the highest ASIR in 1990, but the ASIR of TBL cancer in Bahrain dropped between 1990 and 2019. Palestine, Turkey, and Lebanon were the three top countries in ASIR in 2019. Except for Bahrain and Kuwait, all the indices in all the other countries had an increasing trend between 1990 and 2019 in women. Egypt had the lowest ASIR, ASDR, ASPR, and age-standardized DALY rate in 1990; however, despite many tobacco control attempts (10), all the indices increased between 1990 and 2019. The poor healthcare system, late diagnosis, high prevalence of tobacco smoking, more efficient cancer registration system, unhealthy nutritional pattern, and weak tobacco policies in Lebanon and Turkey are among the reasons leading to a high incidence of TLB cancer in these countries (33–35). In addition, the prevalence of tobacco use among the adolescents aged 13–15 years has recently increased in the countries of the region (36). The smoking rate and air pollution in Egypt have increased in the last decades leading to an increase in TBL cancer incidence. Effective educational health programs, antismoking media messaging, worksite smoking bans, warning labels, etc., could contribute to tobacco cessation among the Egyptian population (37, 38). The World Health Organization’s (WHO) Framework Convention on Tobacco Control has been implemented in Bahrain and several other countries like Algeria, but its implementation and application remain insufficient. It seems that quit tobacco clinics (QTC) and other smoking cessation programs, such as sponsorship and advertisement ban, increasing cigarette taxes, media campaigns, and nicotine replacement therapy, have succeeded in decreasing tobacco smoking in this country (12, 39, 40). The decreased rate of ASIR in men and women could be attributed to these campaigns and interventions. Lung squamous cell carcinoma that is highly associated with smoking is decreasing in Bahrain, showing the crucial role of the decline in smoking in the occurrence of TBL cancer (41). Moreover, the rapid economic development of Kuwait and all the mentioned factors for Bahrain have contributed to diminishing tobacco smoking in these countries (42, 43).

Overall, ASDR, DALYs, YLLs, and YLDs attributable to all risk factors of TBL cancer decreased between 1990 and 2019. Improvement of healthcare systems in diagnosis and treatment of cancers has led to a decrease in YLLs and DALYs of TBL cancer from 1990 to 2019. Nevertheless, unlike men, attributed ASDR, DALYs, YLLs, and YLDs to risk factors rose in women. Nowadays, women have more exposure to occupational risks and smoke more tobacco, which can explain the increasing trend of attributed risk to death and DALY rate of TBL cancer (27, 29). Tobacco, air pollution, occupational risks, high fasting plasma glucose, residential radon, and a diet low in fruits were the main TBL cancer risk factors. All the risk factors, except high fasting plasma glucose, generally had a decreasing trend in the NAME region countries. Tobacco smoking, the most important risk factor of TBL cancer, is very prevalent in the NAME region and is growing rapidly in women (10). According to the WHO global report on trends in tobacco smoking prevalence from 2000 to 2025 (44), if tobacco control plans in the NAME region are not applied properly, the prevalence of smoking would increase and cause more diseases, such as TBL cancer. Air pollution is also one of the main problems in the NAME region and seems to increase non-smoker TBL cancer (45). Democratic development of the NAME countries could help to lessen the environmental problems (46). Additionally, the incidence of diabetes, another risk factor of TBL cancer, is increasing globally; however, genetic factors, sedentary lifestyle, health illiteracy, inadequate healthcare quality, smoking, demographic, and economic changes in the NAME region have led to a more rapid increase in diabetes prevalence in this region (47, 48).

The incidence and prevalence of TBL cancer, a leading cause of death worldwide, are still increasing in the NAME region. Since TBL cancer is a preventable disease, management and interventions by the healthcare systems could play a crucial role in decreasing its incidence. In addition, the early diagnosis of this cancer should be prioritized to improve the prognosis of TBL cancer. Tobacco continues to be a health concern in this region due to its high prevalence and the increasing trend in women. The region countries are heterogeneous regarding the political stability, SDI, population, or conflict status. Hence, educational policies regarding prevention and risk factors of TBL cancer for people and country-specific intervention could contribute to decreasing the disease burden of TBL cancer in the NAME region (10, 12, 27).

GBD updates its data and allows policymakers to observe the disease burden over time to evaluate the impact of health control programs and monitor country-specific data. However, some limitations should be noted. Combined burden of three causes of cancer as TBL is one of the main limitations of this study. Lack of pathological classification of TBL cancer, low quality of several data sources in several countries, primary data availability, inconsistency of mortality rates, cofounders, challenges with full representation of UIs, collinearity problem in models, mediation bias in risk factors, and sparse data of several risk factors are among our study’s limitations. In addition, some data were not collected using the preferred measurement methods or case definition, and some important determinants, such as social determinants, are not included in the risk factors yet. Adding pathological classification of TBL cancer in future could also improve the quality of GBD data. The strength of our study is that it provides the most recent evaluation of death rates, DALYs, incidence rate, etc., of a disease and its related risk factors. To the best of our knowledge, our study is the first most comprehensive study on the risk factors and burden of TBL in the NAME region and its countries. GBD 2019 used dose–response meta-regressions to determine more accurate results for risk exposure. Many other models were used to report a precise estimation of the disease burden and attributable risk factors. In addition, new data sources and risk factors were added compared with GBD 2016 (49).

## 5 Conclusion

The incidence, prevalence, and YLD rates of TBL cancer increased, and the death rate remained unchanged from 1990 to 2019 in the NAME region. It should be mentioned that all the indices decreased in men but increased in women. The contribution of risk factors decreased in men but increased in women. Smoking is still the main risk factor for TBL cancer. Early diagnosis and tobacco cessation programs should be improved in the NAME region. The GBD TBL cancer estimates can be used to improve the health condition in each country of the NAME region and contribute to establishing effective policies in TBL cancer control.

## Data availability statement

The raw data supporting the conclusions of this article will be made available by the authors, without undue reservation.

## GBD 2019 NAME Tracheal, Bronchus, and Lung Cancer Collaborators

Shaghayegh Khanmohammadi, Non-Communicable Diseases Research Center, Endocrinology and Metabolism Population Sciences Institute, Tehran University of Medical Sciences, Tehran, Iran; School of Medicine, Tehran University of Medical Sciences, Tehran, Iran; Sahar Saeedi Moghaddam, Non-Communicable Diseases Research Center, Endocrinology and Metabolism Population Sciences Institute, Tehran University of Medical Sciences, Tehran, Iran; Sina Azadnajafabad, Non-Communicable Diseases Research Center, Endocrinology and Metabolism Population Sciences Institute, Tehran University of Medical Sciences, Tehran, Iran; Negar Rezaei, Non-Communicable Diseases Research Center, Endocrinology and Metabolism Population Sciences Institute, Tehran University of Medical Sciences, Tehran, Iran; Endocrinology and Metabolism Research Center, Endocrinology and Metabolism Clinical Sciences Institute, Tehran University of Medical Sciences, Tehran, Iran; Zahra Esfahani, Non-Communicable Diseases Research Center, Endocrinology and Metabolism Population Sciences Institute, Tehran University of Medical Sciences, Tehran, Iran; Nazila Rezaei, Non-Communicable Diseases Research Center, Endocrinology and Metabolism Population Sciences Institute, Tehran University of Medical Sciences, Tehran, Iran; Mohsen Abbasi-Kangevari, Non-Communicable Diseases Research Center, Endocrinology and Metabolism Population Sciences Institute, Tehran University of Medical Sciences, Tehran, Iran; Zeinab Abbasi-Kangevari, Non-Communicable Diseases Research Center, Endocrinology and Metabolism Population Sciences Institute, Tehran University of Medical Sciences, Tehran, Iran; Meriem Abdoun, Department of Medicine, University of Setif Algeria; Sétif, Algeria Hassan Abidi, Laboratory Technology Sciences Department, Yasuj University of Medical Sciences, Yasuj, Iran; Zahra Abrehdari-Tafreshi, Cellular and Molecular Biology Department, University of Tehran, Tehran, Iran; Ahmed Abu-Zaid, Department of Surgery, Obstetrics & Gynecology, Alfaisal University, Riyadh, Saudi Arabia; College of Graduate Health Sciences, University of Tennessee, Memphis, TN, United States; Aqeel Ahmad, Department of Medical Biochemistry, Shaqra University, Shaqra, Saudi Arabia; Sepideh Ahmadi, School of Advanced Technologies in Medicine, Shahid Beheshti University of Medical Sciences, Tehran, Iran; Hanadi Al Hamad, Geriatric and Long Term Care Department, Hamad Medical Corporation, Doha, Qatar; Rumailah Hospital, Hamad Medical Corporation, Doha, Qatar; Saleh Ali Alessy, Department of Public Health, Saudi Electronic University, Riyadh, Saudi Arabia; Centre for Cancer, Society & Public Health, King’s College London, London, United Kingdom; Syed Mohamed Aljunid, Department of Health Policy and Management, Kuwait University, Kuwait, Kuwait; International Centre for Casemix and Clinical Coding, National University of Malaysia, Bandar Tun Razak, Malaysia; Mehrdad Amir-Behghadami, Road Traffic Injury Research Center, Tabriz University of Medical Sciences, Tabriz, Iran; Department of Health Service Management, Iranian Center of Excellence in Health Management, Tabriz, Iran; Alireza Ansari-Moghaddam, Department of Epidemiology and Biostatistics, Zahedan University of Medical Sciences, Zahedan, Iran; Jalal Arabloo, Health Management and Economics Research Center, Iran University of Medical Sciences, Tehran, Iran; Mohammadreza Azangou-Khyavy, Non-Communicable Diseases Research Center, Endocrinology and Metabolism Population Sciences Institute, Tehran University of Medical Sciences, Tehran, Iran; Social Determinants of Health Research Center, Shahid Beheshti University of Medical Sciences, Tehran, Iran; Nayereh Baghcheghi, Department of Nursing, Saveh University of Medical Sciences, saveh, Iran; Khuloud Bajbouj, Department of Basic Medical Sciences, University of Sharjah, Sharjah, United Arab Emirates; Ali Bijani, Social Determinants of Health Research Center, Babol University of Medical Sciences, Babol, Iran; Mariah Malak Bilalaga, Department of Clinical Sciences, University of Sharjah, Sharjah, United Arab Emirates; Souad Bouaoud, Department of Medicine, Faculty of Medicine University Farhat Abbas, Setif, Algeria; Department of Epidemiology and Preventive Medicine, University Hospital Saadna Abdenour, Setif, Algeria; Daniela Calina, Department of Clinical Pharmacy, University of Medicine and Pharmacy of Craiova, Romania, Craiova, Romania; William C S Cho, Department of Clinical Oncology, Queen Elizabeth Hospital, Hong Kong, Hong Kong SAR, China; Omar B Da’ar, Department of Health Systems Management, King Saud bin Abdulaziz University for Health Sciences, Riyadh, Saudi Arabia; Shirin Djalalinia, Development of Research and Technology Center, Ministry of Health and Medical Education, Tehran, Iran; Hesham Elghazaly, Department of Oncology, Ain Shams University, Cairo, Egypt; Department of Clinical Research, Faculty of Medicine Ain Shams Research Institute (MASRI), Cairo, Egypt; Muhammed Elhadi, Faculty of Medicine, University of Tripoli, Tripoli, Libya; Rana Ezzeddini, Department of Clinical Biochemistry, Tarbiat Modares University, Tehran, Iran; Alireza Feizkhah, Department of Social Medicine and Epidemiology, Guilan University of Medical Sciences, Rasht, Iran; Ahmad Ghashghaee, School of Public Health, Qazvin University of Medical Sciences, Qazvin, Iran; Mohamad Golitaleb, Department of Nursing, Arak University of Medical Sciences, Arak, Iran; Atlas Haddadi Avval, School of Medicine, Mashhad University of Medical Sciences, Mashhad, Iran; Nima Hafezi-Nejad, School of Medicine, Tehran University of Medical Sciences, Tehran, Iran; Department of Radiology and Radiological Science, Johns Hopkins University, Baltimore, MD, United States; Randah R Hamadeh, Department of Family and Community Medicine, Arabian Gulf University, Manama, Bahrain; Mahsa Jalili, Department of Microbiology, Hamadan University of Medical Sciences, Hamadan, Iran; Elham Jamshidi, Functional Neurosurgery Research Center, Shahid Beheshti University of Medical Sciences, Tehran, Iran; Division of Pulmonary Medicine, Lausanne University Hospital (CHUV), Lausanne, Switzerland; Amirali Karimi, School of Medicine, Tehran University of Medical Sciences, Tehran, Iran; Yousef Saleh Khader, Department of Public Health, Jordan University of Science and Technology, Irbid, Jordan; Javad Khanali, Non-Communicable Diseases Research Center, Endocrinology and Metabolism Population Sciences Institute, Tehran University of Medical Sciences, Tehran, Iran; Social Determinants of Health Research Center, Shahid Beheshti University of Medical Sciences, Tehran, Iran; Farzad Kompani, Children’s Medical Center, Tehran University of Medical Sciences, Tehran, Iran; Hamid Reza Koohestani, Social Determinants of Health Research Center, Saveh University of Medical Sciences, Saveh, Iran; Burcu Kucuk Bicer, Faculty of Medicine, Gazi University, Ankara, Türkiye; Ahmad R Mafi, Department of Clinical Oncology, Shahid Beheshti University of Medical Sciences, Tehran, Iran; Ata Mahmoodpoor, Department of Anesthesiology and Critical Care, Tabriz University of Medical Sciences, Tabriz, Iran; Mohammad-Reza Malekpour, Non-Communicable Diseases Research Center, Endocrinology and Metabolism Population Sciences Institute, Tehran University of Medical Sciences, Tehran, Iran; Ahmad Azam Malik, Rabigh Faculty of Medicine, King Abdulaziz University, Jeddah, Saudi Arabia; University Institute of Public Health, The University of Lahore, Lahore, Pakistan; Reza Mirfakhraie, Department of Genetics, Shahid Beheshti University of Medical Sciences, Tehran, Iran; Esmaeil Mohammadi, Non-Communicable Diseases Research Center, Endocrinology and Metabolism Population Sciences Institute, Tehran University of Medical Sciences, Tehran, Iran; School of Medicine, Tehran University of Medical Sciences, Tehran, Iran; Sara Momtazmanesh, Non-Communicable Diseases Research Center, Endocrinology and Metabolism Population Sciences Institute, Tehran University of Medical Sciences, Tehran, Iran; School of Medicine, Tehran University of Medical Sciences, Tehran, Iran; Rahmatollah Moradzadeh, Department of Epidemiology, Arak University of Medical Sciences, Arak, Iran; Paula Moraga, Computer, Electrical, and Mathematical Sciences and Engineering Division, King Abdullah University of Science and Technology, Thuwal, Saudi Arabia; Zuhair S Natto, Department of Dental Public Health, King Abdulaziz University, Jeddah, Saudi Arabia; Department of Health Policy and Oral Epidemiology, Harvard University, Boston, MA, United States; Maryam Noori, Student Research Committee, Iran University of Medical Sciences, Tehran, Iran; Simone Perna, Department of Biology, University of Bahrain, Sakir, Bahrain; Raffaele Pezzani, Department of Medicine, Endocrinology Unit, University of Padova, Padova, Italy; AIROB (Associazione Italiana Ricerca Oncologica di Base), Padova, Italy; Majid Pirestani, Department of Parasitology and Entomology, Tarbiat Modares University, Tehran, Iran; Ashkan Pourabhari Langroudi, Non-Communicable Diseases Research Center, Endocrinology and Metabolism Population Sciences Institute, Tehran University of Medical Sciences, Tehran, Iran; Mohammad Rabiee, Biomedical Engineering Department, Amirkabir University of Technology, Tehran, Iran; Navid Rabiee, School of Engineering, Macquarie University, Sydney, NSW, Australia; Pohang University of Science and Technology, Pohang, South Korea; Shayan Rahmani, Non-Communicable Diseases Research Center, Endocrinology and Metabolism Population Sciences Institute, Tehran University of Medical Sciences, Tehran, Iran; Student Research Committee, Shahid Beheshti University of Medical Sciences, Tehran, Iran; Elrashdy Moustafa Mohamed Redwan, Department of Biological Sciences, King Abdulaziz University, Jeddah, Egypt; Department of Protein Research, Research and Academic Institution, Alexandria, Egypt; Nima Rezaei, Research Center for Immunodeficiencies, Tehran University of Medical Sciences, Tehran, Iran; Network of Immunity in Infection, Malignancy and Autoimmunity (NIIMA), Universal Scientific Education and Research Network (USERN), Tehran, Iran; Gholamreza Roshandel, Golestan Research Center of Gastroenterology and Hepatology, Golestan University of Medical Sciences, Gorgan, Iran; Erfan Sadeghi, Department of Biostatistics and Epidemiology, Isfahan University of Medical Sciences, Isfahan, Iran; Amir Salek Farrokhi, Department of Immunology, Semnan University of Medical Sciences and Health Services, Semnan, Iran; Abdallah M Samy, Department of Entomology, Ain Shams University, Cairo, Egypt; Brijesh Sathian, Geriatric and Long Term Care Department, Hamad Medical Corporation, Doha, Qatar; Faculty of Health and Social Sciences, Bournemouth University, Bournemouth, United Kingdom; Saeed Shahabi, Health Policy Research Center, Shiraz University of Medical Sciences, Shiraz, Iran; Javad Sharifi-Rad, Faculty of Medicine, Facultad de Medicina, Universidad del Azuay (Faculty of Medicine, University of Azuay), Cuenca, Ecuador; Sara Sheikhbahaei, Department of Radiology and Radiological Science, Johns Hopkins University, Baltimore, MD, United States; Zahra Shokri Varniab, Non-Communicable Diseases Research Center, Endocrinology and Metabolism Population Sciences Institute, Tehran University of Medical Sciences, Tehran, Iran; Seyed Afshin Shorofi, Department of Medical-Surgical Nursing, Mazandaran University of Medical Sciences, Sari, Iran; Department of Nursing and Health Sciences, Flinders University, Adelaide, SA, Australia; Moslem Taheri Soodejani , Department of Biostatistics and Epidemiology, Shahid Sadoughi University of Medical Sciences, Yazd, Iran; Abdelghani Tbakhi, Department of Cell Therapy and Applied Genomics, King Hussein Cancer Center, Amman, Jordan; Arash Tehrani-Banihashemi, Preventive Medicine and Public Health Research Center, Iran University of Medical Sciences, Tehran, Iran; Department of Community and Family Medicine, Iran University of Medical Sciences, Tehran, Iran; Sahel Valadan Tahbaz, Clinical Cancer Research Center, Milad General Hospital, Tehran, Iran; Department of Microbiology, Islamic Azad University, Tehran, Iran; Seyed Hossein Yahyazadeh Jabbari, Clinical Cancer Research Center, Milad General Hospital, Tehran, Iran; Zabihollah Yousefi, Department of Environmental Health, Mazandaran University of Medical Sciences, Sari, Iran; Maryam Zamanian, Department of Epidemiology, Arak University of Medical Sciences, Arak, Iran; Iman Zare, Research and Development Department, Sina Medical Biochemistry Technologies, Shiraz, Iran; Armin Zarrintan, Department of Radiology, Tabriz University of Medical Sciences, Tabriz, Iran; Mohammad Zoladl, Department of Nursing, Yasuj University of Medical Sciences, Yasuj, Iran; Mohsen Naghavi, Institute for Health Metrics and Evaluation, University of Washington, Seattle, WA, United States; Department of Health Metrics Sciences, School of Medicine, University of Washington, Seattle, WA, United States; Bagher Larijani, Endocrinology and Metabolism Research Center, Endocrinology and Metabolism Clinical Sciences Institute, Tehran University of Medical Sciences, Tehran, Iran; Farshad Farzadfar, Non-Communicable Diseases Research Center, Endocrinology and Metabolism Population Sciences Institute, Tehran University of Medical Sciences, Tehran, Iran; Endocrinology and Metabolism Research Center, Endocrinology and Metabolism Clinical Sciences Institute, Tehran University of Medical Sciences, Tehran, Iran.

## Author contributions

Please see the Appendix for more detailed information about individual author contributions to the research, divided into the following categories: providing data or critical feedback on data sources; developing methods or computational machinery; providing critical feedback on methods or results; drafting the manuscript or revising it critically for important intellectual content; and managing the estimation or publications process.

## Supplementary material

Supplementary Table 1 Data used for TBL cancer in the NAME region.

Supplementary Table 2 Decomposition analysis of new cases change cause by sex in countries of the region, between 1990 and 2019.

Supplementary Table 3 Age-standardized rate (per 100,000) of incidence, prevalence, deaths, DALYs, YLDs, and YLLs in counties of the region by sex in 1990 and 2019 with their percent changes YLLs, years of life lost; YLDs, years lived with disability; DALYs, disability-adjusted life years.

Supplementary Table 4 Attributed age-standardized rate (per 100,000) of deaths, DALYs, YLDs, and YLLs to all risk factors in countries of the region by sex in 1990 and 2019 with their percent changes YLLs, years of life lost; YLDs, years lived with disability; DALYs, disability-adjusted life years.

Supplementary Figure The ranking of age-standardized attributed burden to risk factors at the NAME region and its countries, 2019 **(A)** deaths, **(B)** DALYs.

## Appendix

Providing data or critical feedback on data sources

Meriem Abdoun, Hassan Abidi, Ahmed Abu-Zaid, Sepideh Ahmadi, Hanadi Al Hamad, Syed Mohamed Aljunid, Mehrdad Amir-Behghadami, Jalal Arabloo, Khuloud Bajbouj, Souad Bouaoud, Daniela Calina, William C S Cho, Shirin Djalalinia, Hesham Elghazaly, Zahra Esfahani, Farshad Farzadfar, Alireza Feizkhah, Ahmad Ghashghaee, Mohamad Golitaleb, Nima Hafezi-Nejad, Mahsa Jalili, Yousef Saleh Khader, Shaghayegh Khanmohammadi, Ahmad R Mafi, Ata Mahmoodpoor, Mohsen Naghavi, Zuhair S Natto, Maryam Noori, Simone Perna, Shayan Rahmani, Nima Rezaei, Sahar Saeedi Moghaddam, Abdallah M Samy, Brijesh Sathian, Javad Sharifi-Rad, Sara Sheikhbahaei, Sahel Valadan Tahbaz, Seyed Hossein Yahyazadeh Jabbari, Iman Zare, and Mohammad Zoladl.

Developing methods or computational machinery

Shaghayegh Khanmohammadi, Mohsen Naghavi, and Sahar Saeedi Moghaddam.

Providing critical feedback on methods or results

Meriem Abdoun, Hassan Abidi, Zahra Abrehdari-Tafreshi, Ahmed Abu-Zaid, Aqeel Ahmad, Hanadi Al Hamad, Saleh Ali Alessy, Syed Mohamed Aljunid, Mehrdad Amir-Behghadami, Alireza Ansari-Moghaddam, Jalal Arabloo, Sina Azadnajafabad, Mohammadreza Azangou-Khyavy, Nayereh Baghcheghi, Khuloud Bajbouj, Ali Bijani, Mariah Malak Bilalaga, Souad Bouaoud, Daniela Calina, William C S Cho, Omar B Da'ar, Muhammed Elhadi, Rana Ezzeddini, Farshad Farzadfar, Alireza Feizkhah, Ahmad Ghashghaee, Mohamad Golitaleb, Atlas Haddadi Avval, Nima Hafezi-Nejad, Randah R Hamadeh, Mahsa Jalili, Elham Jamshidi, Amirali Karimi, Yousef Saleh Khader, Javad Khanali, Shaghayegh Khanmohammadi, Farzad Kompani, Hamid Reza Koohestani, Burcu Kucuk Bicer, Bagher Larijani, Ahmad R Mafi, Ata Mahmoodpoor, Mohammad-Reza Malekpour, Ahmad Azam Malik, Reza Mirfakhraie, Esmaeil Mohammadi, Sara Momtazmanesh, Rahmatollah Moradzadeh, Mohsen Naghavi, Zuhair S Natto, Simone Perna, Raffaele Pezzani, Majid Pirestani, Ashkan Pourabhari Langroudi, Navid Rabiee, Shayan Rahmani, Negar Rezaei, Nima Rezaei, Gholamreza Roshandel, Erfan Sadeghi, Sahar Saeedi Moghaddam, Amir Salek Farrokhi, Abdallah M Samy, Brijesh Sathian, Saeed Shahabi, Javad Sharifi-Rad, Sara Sheikhbahaei, Zahra Shokri Varniab, Seyed Afshin Shorofi, Moslem Taheri Soodejani, Abdelghani Tbakhi, Sahel Valadan Tahbaz, Seyed Hossein Yahyazadeh Jabbari, Zabihollah Yousefi, Maryam Zamanian, Armin Zarrintan, and Mohammad Zoladl.

Drafting the work or revising is critically for important intellectual content

Mohsen Abbasi-Kangevari, Zeinab Abbasi-Kangevari, Meriem Abdoun, Hassan Abidi, Ahmed Abu-Zaid, Sepideh Ahmadi, Mehrdad Amir-Behghadami, Jalal Arabloo, Sina Azadnajafabad, Mohammadreza Azangou-Khyavy, Khuloud Bajbouj, Mariah Malak Bilalaga, Daniela Calina, William C S Cho, Hesham Elghazaly, Muhammed Elhadi, Zahra Esfahani, Rana Ezzeddini, Farshad Farzadfar, Alireza Feizkhah, Mohamad Golitaleb, Atlas Haddadi Avval, Nima Hafezi-Nejad, Randah R Hamadeh, Mahsa Jalili, Yousef Saleh Khader, Javad Khanali, Shaghayegh Khanmohammadi, Burcu Kucuk Bicer, Bagher Larijani, Mohammad-Reza Malekpour, Ahmad Azam Malik, Reza Mirfakhraie, Sara Momtazmanesh, Paula Moraga, Mohsen Naghavi, Zuhair S Natto, Simone Perna, Ashkan Pourabhari Langroudi, Mohammad Rabiee, Navid Rabiee, Shayan Rahmani, Elrashdy Moustafa Mohamed Redwan, Nazila Rezaei, Negar Rezaei, Nima Rezaei, Sahar Saeedi Moghaddam, Amir Salek Farrokhi, Abdallah M Samy, Saeed Shahabi, Javad Sharifi-Rad, Sara Sheikhbahaei, Seyed Afshin Shorofi, Arash Tehrani-Banihashemi, Sahel Valadan Tahbaz, Seyed Hossein Yahyazadeh Jabbari, Maryam Zamanian, Iman Zare, Armin Zarrintan, and Mohammad Zoladl.

Managing the estimation or publications process

Sina Azadnajafabad, Shaghayegh Khanmohammadi, Bagher Larijani, Mohsen Naghavi, Nazila Rezaei, and Sahar Saeedi Moghaddam.

## References

[1] Ebrahimi H Aryan Z Saeedi Moghaddam S Bisignano C Rezaei S Pishgar F. Global, regional, and national burden of respiratory tract cancers and associated risk factors from 1990 to 2019: A systematic analysis for the global burden of disease study 2019. Lancet Respir Med (2021) 9(9):1030–49. doi: 10.1016/S2213-2600(21)00164-8 PMC8410610 34411511

[2] Fitzmaurice C Akinyemiju TF Al Lami FH Alam T Alizadeh-Navaei R Allen C. Global, regional, and national cancer incidence, mortality, years of life lost, years lived with disability, and disability-adjusted life-years for 29 cancer groups, 1990 to 2016: A systematic analysis for the global burden of disease study. JAMA Oncol (2018) 4(11):1553–68. doi: 10.1001/jamaoncol.2018.2706 PMC6248091 29860482

[3] Tas F Ciftci R Kilic L Karabulut S. Age is a prognostic factor affecting survival in lung cancer patients. Oncol Lett (2013) 6(5):1507–13. doi: 10.3892/ol.2013.1566 PMC3813578 24179550

[4] Essam A El-Moselhy AWE. Risk factors of lung cancer worldwide and in Egypt: Current situation. J Oncopathol Clin Res (2018) 2.

[5] Cheng TY Cramb SM Baade PD Youlden DR Nwogu C Reid ME. The international epidemiology of lung cancer: Latest trends, disparities, and tumor characteristics. J Thorac Oncol (2016) 11(10):1653–71. doi: 10.1016/j.jtho.2016.05.021 PMC5512876 27364315

[6] Cicin I Oksuz E Karadurmus N Malhan S Gumus M Yilmaz U. Economic burden of lung cancer in Turkey: A cost of illness study from payer perspective. Health Econ Rev (2021) 11(1):22. doi: 10.1186/s13561-021-00322-2 34173876 PMC8233643

[7] Jeon SM Kwon JW Choi SH Park HY. Economic burden of lung cancer: A retrospective cohort study in south Korea, 2002-2015. PloS One (2019) 14(2):e0212878. doi: 10.1371/journal.pone.0212878 30794674 PMC6386401

[8] Kutikova L Bowman L Chang S Long SR Obasaju C Crown WH. The economic burden of lung cancer and the associated costs of treatment failure in the united states. Lung Cancer (2005) 50(2):143–54. doi: 10.1016/j.lungcan.2005.06.005 16112249

[9] Gelatti ACZ Lorandi V. Challenging scenarios in the treatment of lung cancer. J Bras Pneumol (2020) 46. doi: 10.36416/1806-3756/e20200388 PMC7567615 32901690

[10] Jazieh AR Algwaiz G Errihani H Elghissassi I Mula-Hussain L Bawazir AA. Lung cancer in the middle East and north Africa region. J Thorac Oncol (2019) 14(11):1884–91. doi: 10.1016/j.jtho.2019.02.016 31668315

[11] Boutayeb A Helmert U. Social inequalities, regional disparities and health inequity in North African countries. Int J Equity Health (2011) 10, 23. doi: 10.1186/1475-9276-10-23 21627818 PMC3120653

[12] Hamadeh RR Ahmed J Al-Kawari M Bucheeri S. Quit tobacco clinics in Bahrain: smoking cessation rates and patient satisfaction. Tobacco Induced Dis (2017) 15(1):7. doi: 10.1186/s12971-017-0115-1 PMC5251216 28127273

[13] Salim EI Jazieh AR Moore MA. Lung cancer incidence in the arab league countries: risk factors and control. Asian Pac J Cancer Prev (2011) 12(1):17–34. 21517227

[14] Alessy SA Znaor A Shamseddine A Fouad H Zendehdel K Abdul-Sater Z. Cancer surveillance in the Eastern Mediterranean region. Lung (2022) 51:7.

[15] Hawari FI Bader RK. Advancing tobacco dependence treatment services in the Eastern Mediterranean region: International collaboration for training and capacity-building. Sultan Qaboos Univ Med J (2014) 14(4):e442–7. PMC4205053 25364544

[16] Nagi M Riewpaiboon A Thavorncharoensap M. Cost of premature mortality attributable to smoking in the middle East and north Africa. East Mediterr Health J (2021) 27. doi: 10.26719/emhj.21.028 34766323

[17] Murray CJL Aravkin AY Zheng P Abbafati C Abbas KM Abbasi-Kangevari M. Global burden of 87 risk factors in 204 countries and territories, 1990-2019: a systematic analysis for the global burden of disease study 2019. Lancet (2020) 396(10258):1223–49. doi: 10.1016/S0140-6736(20)30752-2 PMC7566194 33069327

[18] Vos T Lim SS Abbafati C Abbas KM Abbasi M Abbasifard M. Global burden of 369 diseases and injuries in 204 countries and territories, 1990-2019: a systematic analysis for the global burden of disease study 2019. Lancet (2020) 396(10258):1204–22. doi: 10.1016/S0140-6736(20)30925-9 PMC7567026 33069326

[19] Foreman KJ Lozano R Lopez AD Murray CJ. Modeling causes of death: an integrated approach using CODEm. Popul Health Metr (2012) 10:1. doi: 10.1186/1478-7954-10-1 22226226 PMC3315398

[20] Global burden of disease study 2019 (GBD 2019) cause list mapped to ICD codes: IHME (2020). Available at: http://ghdx.healthdata.org/record/ihme-data/gbd-2019-cause-icd-code-mappings.

[21] Murray CJL Ezzati M Lopez AD Rodgers A Vander Hoorn S. Comparative quantification of health risks conceptual framework and methodological issues. Popul Health Metr (2003) 1(1):1–. doi: 10.1186/1478-7954-1-1 PMC156894 12780936

[22] Wang H Abbas KM Abbasifard M Abbasi-Kangevari M Abbastabar H Abd-Allah F. Global age-sex-specific fertility, mortality, healthy life expectancy (HALE), and population estimates in 204 countries and territories, 1950-2019: a comprehensive demographic analysis for the global burden of disease study 2019. Lancet (2020) 396(10258):1160–203. doi: 10.1016/S0140-6736(20)30977-6 PMC7566045 33069325

[23] Gupta PD. Standardization and decomposition of rates: A user's manual: Census (1993). Available at: https://www.census.gov/library/publications/1993/demo/p23-186.html.

[24] Fitzmaurice C Allen C Barber RM Barregard L Bhutta ZA Brenner H. Global, regional, and national cancer incidence, mortality, years of life lost, years lived with disability, and disability-adjusted life-years for 32 cancer groups, 1990 to 2015: A systematic analysis for the global burden of disease study. JAMA Oncol (2017) 3(4):524–48. doi: 10.1001/jamaoncol.2016.5688 PMC6103527 27918777

[25] Wong MCS Lao XQ Ho K-F Goggins WB Tse SLA. Incidence and mortality of lung cancer: Global trends and association with socioeconomic status. Sci Rep (2017) 7(1):14300. doi: 10.1038/s41598-017-14513-7 29085026 PMC5662733

[26] Zhou B Zang R Zhang M Song P Liu L Bie F. Worldwide burden and epidemiological trends of tracheal, bronchus, and lung cancer: A population-based study. EBioMedicine (2022) 78:103951. doi: 10.1016/j.ebiom.2022.103951 35313216 PMC8935504

[27] Khattab A Javaid A Iraqi G Alzaabi A Ben Kheder A Koniski M-L. Smoking habits in the middle East and north Africa: Results of the BREATHE study. Respir Med (2012) 106:S16–24. doi: 10.1016/S0954-6111(12)70011-2 23290700

[28] Deng Y Zhao P Zhou L Xiang D Hu J Liu Y. Epidemiological trends of tracheal, bronchus, and lung cancer at the global, regional, and national levels: a population-based study. J Hematol Oncol (2020) 13(1):98. doi: 10.1186/s13045-020-00915-0 32690044 PMC7370495

[29] Allen AM Oncken C Hatsukami D. Women and smoking: The effect of gender on the epidemiology, health effects, and cessation of smoking. Curr Addict Rep (2014) 1(1):53–60. doi: 10.1007/s40429-013-0003-6 27213132 PMC4871621

[30] Alzaabi A Mahboub B Salhi H Kajingu W Rashid N El-Hasnaoui A. Waterpipe use in the middle East and north Africa: Data from the breathe study. Nicotine Tob Res (2017) 19(11):1375–80. doi: 10.1093/ntr/ntw256 29017260

[31] Masjedi MR Naghan PA Taslimi S Yousefifard M Ebrahimi SM Khosravi A. Opium could be considered an independent risk factor for lung cancer: A case-control study. Respiration (2013) 85(2):112–8. doi: 10.1159/000338559 22759984

[32] Gildea TR DaCosta Byfield S Hogarth DK Wilson DS Quinn CC. A retrospective analysis of delays in the diagnosis of lung cancer and associated costs. Clinicoecon Outcomes Res (2017) 9:261–9. doi: 10.2147/CEOR.S132259 PMC5440037 28553128

[33] Salhab HA Fares MY Khachfe HH Khachfe HM. Epidemiological study of lung cancer incidence in Lebanon. Med (Kaunas) (2019) 55(6). doi: 10.3390/medicina55060217 PMC6631477 31141934

[34] Kansu S Koparal B Kirdar L Batirel H Çetingöz R Özet A. PA01.05 lung cancer management in Turkey. J Thorac Oncol (2017) 12(1):S212–S3. doi: 10.1016/j.jtho.2016.11.190

[35] Rania Abu Seir AK Ghannam I. Prevalence of tobacco use among young adults in Palestine. EMHJ (2020) 26(1). doi: 10.26719/2020.26.1.75 32043549

[36] Ma C Xi B Li Z Wu H Zhao M Liang Y. Prevalence and trends in tobacco use among adolescents aged 13–15 years in 143 countries, 1999–2018: findings from the global youth tobacco surveys. Lancet Child Adolesc Health (2021) 5(4):245–55. doi: 10.1016/S2352-4642(20)30390-4 33545071

[37] Fouda S Kelany M Moustafa N Abushouk AI Hassane A Sleem A. Tobacco smoking in Egypt: A scoping literature review of its epidemiology and control measures. East Mediterr Health J (2018) 24(2):198–215. doi: 10.26719/2018.24.2.198 29748949

[38] Safar ZS Labib MW. Assessment of particulate matter and lead levels in the greater Cairo area for the period 1998–2007. J Adv Res (2010) 1(1):53–63. doi: 10.1016/j.jare.2010.02.004

[39] Hamadeh RR Ahmed J Al Kawari M Bucheeri S. Smoking behavior of males attending the quit tobacco clinics in Bahrain and their knowledge on tobacco smoking health hazards. BMC Public Health (2018) 18(1):199. doi: 10.1186/s12889-018-5104-7 29378543 PMC5789601

[40] WHO. Effects of meeting MPOWER requirements on smoking rates and smoking-attributable deaths: Bahrain. (2018).

[41] Mubarak A Aljufairi E Almahari S. Lung cancer in Bahrain: Histological and molecular features. Gulf J Oncol (2020) 1:48–51. 33431362

[42] WHO. Effects of meeting MPOWER requirements on smoking rates and smoking-attributable deaths: Kuwait. (2018).

[43] Alali WQ Longenecker JC Alwotyan R AlKandari H Al-Mulla F Al Duwairi Q. Prevalence of smoking in the Kuwaiti adult population in 2014: A cross-sectional study. Environ Sci pollut Res (2021) 28(8):10053–67. doi: 10.1007/s11356-020-11464-x PMC7648895 33161520

[44] WHO. WHO global report on trends in prevalence of tobacco smoking 2000-2025. (2018). Springer, Cham

[45] Khanjani N. Air pollution and its health effects in the Eastern Mediterranean region.

[46] Farzanegan MR Markwardt G. Development and pollution in the middle East and north Africa: Democracy matters. J Policy Model (2018) 40(2):350–74. doi: 10.1016/j.jpolmod.2018.01.010

[47] Khan Y Hamdy O. Type 2 diabetes in the middle east and north Africa (MENA). (2017), 49–61. doi: 10.1007/978-3-319-41559-8_4

[48] Al Busaidi N Shanmugam P Manoharan D. Diabetes in the middle East: Government health care policies and strategies that address the growing diabetes prevalence in the middle East. Curr Diabetes Rep (2019) 19(2):8. doi: 10.1007/s11892-019-1125-6 30715611

[49] Vos T Abajobir AA Abbafati C Abbas KM Abate KH Abd-Allah F. Global, regional, and national incidence, prevalence, and years lived with disability for 328 diseases and injuries for 195 countries, 1990-2016: a systematic analysis for the global burden of disease study 2016. Lancet (2017) 390(10100):1211–59. doi: 10.1016/S0140-6736(17)32154-2 PMC5605509 28919117

