# Erratum: Burden of tracheal, bronchus, and lung cancer in North Africa and Middle East countries, 1990 to 2019: results from the GBD study 2019

**DOI:** 10.3389/fonc.2023.1253486

**Published:** 2023-07-18

**Authors:** Frontiers Production Office

**Affiliations:** Frontiers Media SA, Lausanne, Switzerland

**Keywords:** tracheal cancer, bronchus cancer, lung neoplasms, global burden of disease, attributable risks, tobacco use, incidence, death

Due to a production error, there was a mistake in the author list. In the published article, the GBD 2019 NAME Tracheal, Bronchus, and Lung Cancer Collaborators were not individually credited as authors in the XML tagging.

The publisher apologizes for this mistake. The original version of this article has been updated.

