# Supplementary material for: Burden of tracheal, bronchus, and lung cancer in North Africa and Middle East countries, 1990 to 2019: Results from the GBD study 2019
Source: Front Oncol. 2023 Feb 10;12:1098218. doi: 10.3389/fonc.2022.1098218 (PMC9951096; doi:10.3389/fonc.2022.1098218)
Supplement: Supplementary Table 1 — Data used for TBL cancer in the NAME region. [file DataSheet_1.pdf]

Supplementary table 1

**Suggested citation**
Aden Cancer Registry and Research Centre (Yemen). Yemen Cancer Incidence Report 2007-2011. Aden, Yemen: Aden Cancer Registry and Research Centre (Yemen), 2013.
Al-Tarawneh M, Khatib S, Arqub K. Cancer incidence in Jordan, 1996-2005. East Mediterr Health J. 2010; 16(8): 837-45.
Ardabil University of Medical Sciences, Digestive Diseases Research Center (Iran), International Agency for Research on Cancer (IARC). Iran - Ardabil Cancer Registry Extracts 1985-2008.
Cancer Control Department, Ministry of Health (Turkey). Turkey - Antalya Cancer Incidence 2004.
Cancer Control Department, Ministry of Health (Turkey). Turkey - Antalya Cancer Incidence 2008.
Cancer Control Department, Ministry of Health (Turkey). Turkey - Izmir Cancer Incidence 2008.
Cancer Control Department, Ministry of Health (Turkey). Turkey Active Cancer Registration System 8 Provinces Incidence 2007.
Cancer Control Department, Ministry of Health (Turkey). Turkey Active Cancer Registration System 9 Provinces Incidence 2008.
Cancer Control Department, Ministry of Health (Turkey). Turkey Cancer Statistics 2002-2003. Ankara, Turkey: Cancer Control Department, Ministry of Health (Turkey).
Cancer Control Department, Ministry of Health (Turkey). Turkey Cancer Statistics 2004. Ankara, Turkey: Cancer Control Department, Ministry of Health (Turkey).
Cancer Control Department, Ministry of Health (Turkey). Turkey Cancer Statistics 2005. Ankara, Turkey: Cancer Control Department, Ministry of Health (Turkey).
Casablanca Ministry of Health (Morocco), Faculty of Medicine and Pharmacy of Casablanca (Morocco), Ibn Rochd University Hospital (Morocco), Lalla Salma Association to Fight Against Cancer (Morocco), National Institute of Oncology Sidi Mohamed Ben Abdellah Rabat (Morocco). Morocco - Cancer Registry of Greater
Casablanca Ministry of Health (Morocco), Faculty of Medicine and Pharmacy of Casablanca (Morocco), Ibn Rochd University Hospital (Morocco), Lalla Salma Association to Fight Against Cancer (Morocco). Morocco - Cancer Registry of Greater Casablanca Region 2005 2006 2007. Rabat, Morocco: Lalla Salma Association to
Center for Disease Control and Prevention, Ministry of Health and Medical Education (Iran), Digestive Diseases Research Center (Iran), Golestan University of Medical Sciences (Iran). Iran - Golestan Cancer Registry Incidence Data 2006-2008.
Center for Disease Control and Prevention, Ministry of Health and Medical Education (Iran). Iran National Cancer Registry 2000.

Suggested citation

Center for Disease Control and Prevention, Ministry of Health and Medical Education (Iran). Iran National Cancer Registry 2001.
Center for Disease Control and Prevention, Ministry of Health and Medical Education (Iran). Iran National Cancer Registry 2002.
Center for Disease Control and Prevention, Ministry of Health and Medical Education (Iran). Iran National Cancer Registry 2003.
Center for Disease Control and Prevention, Ministry of Health and Medical Education (Iran). Iran National Cancer Registry 2004.
Center for Disease Control and Prevention, Ministry of Health and Medical Education (Iran). Iran National Cancer Registry 2005.
Center for Disease Control and Prevention, Ministry of Health and Medical Education (Iran). Iran National Cancer Registry 2006.
Center for Disease Control and Prevention, Ministry of Health and Medical Education (Iran). Iran National Cancer Registry 2007.
Center for Disease Control and Prevention, Ministry of Health and Medical Education (Iran). Iran National Cancer Registry 2008.
Center for Disease Control and Prevention, Ministry of Health and Medical Education (Iran). Iran National Cancer Registry 2009.
Center for Disease Control and Prevention, Ministry of Health and Medical Education (Iran). Iran National Cancer Registry 2010.
Center for Disease Control and Prevention, Ministry of Health and Medical Education (Iran). Iran National Cancer Registry Report 2003-2004.
Center for Disease Control and Prevention, Ministry of Health and Medical Education (Iran). Iran National Cancer Registry Report 2005-2006.
Center for Disease Control and Prevention, Ministry of Health and Medical Education (Iran). Iran National Cancer Registry Report 2006-2007.
Center for Disease Control and Prevention, Ministry of Health and Medical Education (Iran). Iran National Cancer Registry Report 2008-2009.
Center for Disease Control and Prevention, Ministry of Health and Medical Education (Iran). Iran National Cancer Registry Report 2009-2010.

Suggested citation

Suggested citation
Central Statistics Organization (Afghanistan), ICF International, Ministry of Public Health (Afghanistan). Afghanistan Demographic and Health Survey 2015-2016. Fairfax, United States of America: ICF International, 2017.
Dubai Department of Health and Medical Services. United Arab Emirates - Dubai Health Statistical Yearbook 2003 . Dubai: Dubai Department of Health and Medical Services.
Dubai Department of Health and Medical Services. United Arab Emirates - Dubai Health Statistical Yearbook 2005. Dubai: Dubai Department of Health and Medical Services.
Dubai Department of Health and Medical Services. United Arab Emirates - Dubai Health Statistical Yearbook 2006. Dubai: Dubai Department of Health and Medical Services.
Dubai Department of Health and Medical Services. United Arab Emirates - Dubai Health Statistical Yearbook 2007. Dubai: Dubai Department of Health and Medical Services.
Elbasmi A, Al-Asfour A, Al-Nesf Y, Al-Awadi A. Cancer in Kuwait: magnitude of the problem. Gulf J Oncol. 2010; 8: 7-14.
El-Minia Cancer Center, Ministry of Communications and Information Technology (Egypt), Ministry of Health and Population (Egypt), National Cancer Registry Program of Egypt. Egypt - El-Minia National Cancer Registry Report 2009. Cairo, Egypt: National Cancer Registry Program of Egypt, 2011.
Epidemiological Transition and Health Impact in North Africa (TAHINA), Ministry of Health and Population (Algeria), National Institute of Public Health (Algeria). Algeria - Study of Causes of Death, TAHINA 2002. Alger, Algeria: National Institute of Public Health (Algeria), 2008.
Hamad Medical Corporation (Qatar), National Health Authority (Qatar). Qatar Annual Health Report 2005. Doha, Qatar: Hamad Medical Corporation (Qatar), 2006.
Hamad Medical Corporation (Qatar), National Health Authority (Qatar). Qatar Annual Health Report 2006. Doha, Qatar: Hamad Medical Corporation (Qatar), 2007.
Hamad Medical Corporation (Qatar), National Health Authority (Qatar). Qatar Annual Health Report 2007. Doha, Qatar: Hamad Medical Corporation (Qatar).
Hamad Medical Corporation (Qatar), Supreme Council of Health (Qatar). Qatar Annual Health Report 2008. Doha, Qatar: Hamad Medical Corporation (Qatar).
Hamad Medical Corporation (Qatar), Supreme Council of Health (Qatar). Qatar Annual Health Report 2009. Doha, Qatar: Hamad Medical Corporation (Qatar).
Hamad Medical Corporation (Qatar), Supreme Council of Health (Qatar). Qatar Annual Health Report 2010. Doha, Qatar: Hamad Medical Corporation (Qatar).
Hamad Medical Corporation (Qatar), Supreme Council of Health (Qatar). Qatar Annual Health Report 2011. Doha, Qatar: Hamad Medical Corporation (Qatar), 2012.

**Suggested citation**
Inal A, Kaplan MA, Kucukoner M, Urakcı Z, kılınc F, Isıkdogan A. Is diabetes mellitus a negative prognostic factor for the treatment of advanced non-small-cell lung cancer?. Rev Port Pneumol. 2014; 20(2): 62–8.
Institute for Health Metrics and Evaluation (IHME), Ministry of Health (Saudi Arabia). Saudi Arabia Health Interview Survey 2013.
Iraqi Cancer Board, Ministry of Health (Iraq). Iraq Cancer Registry 2011. Baghdad, Iraq: Ministry of Health (Iraq).
Izmir Cancer Registry (KIDEM). Turkey - Izmir Cancer Registry Incidence 2006.
Jordan Cancer Registry, Middle East Cancer Consortium, National Cancer Institute (United States). Jordan Cancer Incidence Report 2001. Amman, Jordan: Ministry of Health (Jordan).
Jordan Cancer Registry, Middle East Cancer Consortium, National Cancer Institute (United States). Jordan Cancer Incidence Report 2002. Amman, Jordan: Ministry of Health (Jordan).
Jordan Cancer Registry, Middle East Cancer Consortium, National Cancer Institute (United States). Jordan Cancer Incidence Report 2003. Amman, Jordan: Ministry of Health (Jordan).
Jordan Cancer Registry, Middle East Cancer Consortium, National Cancer Institute (United States). Jordan Cancer Incidence Report 2004. Amman, Jordan: Ministry of Health (Jordan).
Jordan Cancer Registry, Middle East Cancer Consortium, National Cancer Institute (United States). Jordan Cancer Incidence Report 2005. Amman, Jordan: Ministry of Health (Jordan).
Jordan Cancer Registry, Middle East Cancer Consortium, National Cancer Institute (United States). Jordan Cancer Incidence Report 2006. Amman, Jordan: Ministry of Health (Jordan).
Jordan Cancer Registry, Middle East Cancer Consortium, National Cancer Institute (United States). Jordan Cancer Incidence Report 2007. Amman, Jordan: Ministry of Health (Jordan).
Jordan Cancer Registry, Middle East Cancer Consortium, National Cancer Institute (United States). Jordan Cancer Incidence Report 2008. Amman, Jordan: Ministry of Health (Jordan).
Jordan Cancer Registry, Middle East Cancer Consortium, National Cancer Institute (United States). Jordan Cancer Incidence Report 2009. Amman, Jordan: Ministry of Health (Jordan).
Jordan Cancer Registry, Middle East Cancer Consortium, National Cancer Institute (United States). Jordan Cancer Incidence Report 2011. Amman, Jordan: Ministry of Health (Jordan).
Jordan Cancer Registry, Middle East Cancer Consortium, National Cancer Institute (United States). Jordan Cancer Incidence Report 2012. Amman, Jordan: Ministry of Health (Jordan).

Suggested citation
Khader YS, Sharkas GF, Arkoub KH, Alfaqih MA, Nimri OF, Khader AM. The Epidemiology and Trend of Cancer in Jordan, 2000-2013. J Cancer Epidemiol. 2018; 2018: 2937067.
Ministry of Development Planning and Statistics (Qatar), Supreme Council of Health (Qatar). Qatar Vital Statistics Annual Bulletin 2014. Doha, Qatar: Ministry of Development Planning and Statistics (Qatar), 2016.
Ministry of Development Planning and Statistics (Qatar), Supreme Council of Health (Qatar). Qatar Vital Statistics Annual Bulletin 2015. Doha, Qatar: Ministry of Development Planning and Statistics (Qatar), 2016.
Ministry of Development Planning and Statistics (Qatar), Supreme Council of Health (Qatar). Qatar Vital Statistics Annual Bulletin 2016. Doha, Qatar: Ministry of Development Planning and Statistics (Qatar), 2017.
Ministry of Development Planning and Statistics (Qatar), Supreme Council of Health (Qatar). Qatar Vital Statistics Annual Bulletin 2017. Doha, Qatar: Ministry of Development Planning and Statistics (Qatar), 2018.
Ministry of Health (Bahrain). Bahrain Health Statistics 2000. Juffair, Bahrain: Ministry of Health (Bahrain).
Ministry of Health (Morocco). Morocco Health in Figures 2004.
Ministry of Health (Morocco). Morocco Health in Figures 2005.
Ministry of Health (Morocco). Morocco Health in Figures 2006 Edition 2007.
Ministry of Health (Morocco). Morocco Health in Figures 2006.
Ministry of Health (Morocco). Morocco Health in Figures 2007 Edition 2008.
Ministry of Health (Morocco). Morocco Health in Figures 2009.
Ministry of Health (Morocco). Morocco Health in Figures 2010.
Ministry of Health (Morocco). Morocco Health in Figures 2011.
Ministry of Health (Morocco). Morocco Health in Figures 2012. Rabat, Morocco: Ministry of Health (Morocco).

Suggested citation
Ministry of Health (Morocco). Morocco Health in Figures 2013. Rabat, Morocco: Ministry of Health (Morocco).
Ministry of Health (Morocco). Morocco Health in Figures 2014. Rabat, Morocco: Ministry of Health (Morocco).
Ministry of Health (Oman), Oman National Cancer Registry. Cancer Incidence in Oman 2007. Muscat, Oman: Ministry of Health (Oman).
Ministry of Health (Oman), Oman National Cancer Registry. Cancer Incidence in Oman 2013. Muscat, Oman: Ministry of Health (Oman).
Ministry of Health (Oman), Oman National Cancer Registry. Oman - Cancer Incidence in Oman 2001. Muscat, Oman: Ministry of Health (Oman).
Ministry of Health (Oman), Oman National Cancer Registry. Oman - Cancer Incidence in Oman 2002. Muscat, Oman: Ministry of Health (Oman).
Ministry of Health (Oman), Oman National Cancer Registry. Oman - Cancer Incidence in Oman 2003. Muscat, Oman: Ministry of Health (Oman).
Ministry of Health (Oman), Oman National Cancer Registry. Oman - Cancer Incidence in Oman 2004. Muscat, Oman: Ministry of Health (Oman).
Ministry of Health (Oman), Oman National Cancer Registry. Oman - Cancer Incidence in Oman 2005. Muscat, Oman: Ministry of Health (Oman).
Ministry of Health (Oman), Oman National Cancer Registry. Oman - Cancer Incidence in Oman 2006. Muscat, Oman: Ministry of Health (Oman).
Ministry of Health (Oman), Oman National Cancer Registry. Oman - Cancer Incidence in Oman 2008. Muscat, Oman: Ministry of Health (Oman).
Ministry of Health (Oman), Oman National Cancer Registry. Oman - Cancer Incidence in Oman 2011. Muscat, Oman: Ministry of Health (Oman).
Ministry of Health (Oman), Oman National Cancer Registry. Oman - Cancer Incidence in Oman 2012. Muscat, Oman: Ministry of Health (Oman).
Ministry of Health (Oman), Oman National Cancer Registry. Oman - Cancer Incidence in Oman Report of 2009. Muscat, Oman: Ministry of Health (Oman).
Ministry of Health (Oman), Oman National Cancer Registry. Oman - Cancer Incidence in Oman Report of 2010. Muscat, Oman: Ministry of Health (Oman).

Suggested citation
Ministry of Health (Oman). Oman Annual Statistical Report 1989. Muscat, Oman: Ministry of Health (Oman).
Ministry of Health (Oman). Oman Annual Statistical Report 1990. Muscat, Oman: Ministry of Health (Oman).
Ministry of Health (Oman). Oman Annual Statistical Report 1991. Muscat, Oman: Ministry of Health (Oman).
Ministry of Health (Oman). Oman Annual Statistical Report 1992. Muscat, Oman: Ministry of Health (Oman).
Ministry of Health (Oman). Oman Annual Statistical Report 1993. Muscat, Oman: Ministry of Health (Oman).
Ministry of Health (Palestine), Palestinian Central Bureau of Statistics, United Nations Children's Fund (UNICEF), United Nations Population Fund (UNFPA). Palestine Multiple Indicator Cluster Survey 2010. New York, United States of America: United Nations Children's Fund (UNICEF), 2014.
Ministry of Health (Palestine). Palestine Health Annual Report 2018. Nablus, Palestine: Ministry of Health (Palestine), 2019.
Ministry of Health (Palestine). Palestine Health Annual Report 2019. Nablus, Palestine: Ministry of Health (Palestine), 2020.
Ministry of Health (Palestine). Palestine Health Annual Report 2020. Nablus, Palestine: Ministry of Health (Palestine), 2021.
Ministry of Health (Palestine). Palestine Health Status Annual Report 1999. Nablus, Palestine: Ministry of Health (Palestine).
Ministry of Health (Palestine). Palestine Health Status Annual Report 2000. Nablus, Palestine: Ministry of Health (Palestine), 2001.
Ministry of Health (Palestine). Palestine Health Status Annual Report 2001. Nablus, Palestine: Ministry of Health (Palestine), 2002.
Ministry of Health (Palestine). Palestine Health Status Annual Report 2012. Nablus, Palestine: Ministry of Health (Palestine), 2013.
Ministry of Health (Palestine). Palestine Health Status Annual Report 2013. Nablus, Palestine: Ministry of Health (Palestine), 2014.
Ministry of Health (Palestine). Palestine Health Status Annual Report 2014. Nablus, Palestine: Ministry of Health (Palestine), 2015.

Suggested citation
Ministry of Health (Palestine). Palestine Health Status Annual Report 2015. Nablus, Palestine: Ministry of Health (Palestine), 2016.
Ministry of Health (Palestine). Palestine Health Status Annual Report 2016. Nablus, Palestine: Ministry of Health (Palestine), 2016.
Ministry of Health (Palestine). Palestine Health Status Annual Report 2017. Nablus, Palestine: Ministry of Health (Palestine), 2018.
Ministry of Health (Saudi Arabia). Saudi Arabia Health Statistical Yearbook 1999. Riyadh, Saudi Arabia: Ministry of Health (Saudi Arabia).
Ministry of Health (Saudi Arabia). Saudi Arabia Health Statistical Yearbook 2000. Riyadh, Saudi Arabia: Ministry of Health (Saudi Arabia).
Ministry of Health (Saudi Arabia). Saudi Arabia Health Statistical Yearbook 2001. Riyadh, Saudi Arabia: Ministry of Health (Saudi Arabia).
Ministry of Health (Saudi Arabia). Saudi Arabia Health Statistical Yearbook 2010. Riyadh, Saudi Arabia: Ministry of Health (Saudi Arabia).
Ministry of Health (Saudi Arabia). Saudi Arabia Health Statistical Yearbook 2011. Riyadh, Saudi Arabia: Ministry of Health (Saudi Arabia).
Ministry of Health (Saudi Arabia). Saudi Arabia Health Statistical Yearbook 2012. Riyadh, Saudi Arabia: Ministry of Health (Saudi Arabia).
Ministry of Health (Saudi Arabia). Saudi Arabia Health Statistical Yearbook 2013. Riyadh, Saudi Arabia: Ministry of Health (Saudi Arabia).
Ministry of Health (Saudi Arabia). Saudi Arabia Health Statistical Yearbook 2014. Riyadh, Saudi Arabia: Ministry of Health (Saudi Arabia).
Ministry of Health (Saudi Arabia). Saudi Arabia Health Statistical Yearbook 2015. Riyadh, Saudi Arabia: Ministry of Health (Saudi Arabia).
Ministry of Health (Saudi Arabia). Saudi Arabia Health Statistical Yearbook 2016. Riyadh, Saudi Arabia: Ministry of Health (Saudi Arabia).
Ministry of Health (Saudi Arabia). Saudi Arabia Health Statistical Yearbook 2017. Riyadh, Saudi Arabia: Ministry of Health (Saudi Arabia).
Ministry of Health (Saudi Arabia). Saudi Arabia Health Statistical Yearbook 2018. Riyadh, Saudi Arabia: Ministry of Health (Saudi Arabia).

Suggested citation
Ministry of Health (Turkey). Turkey Chronic Diseases and Risk Factors Study 2011.
Ministry of Health (Turkey). Turkey Health at a Glance 2007. Ankara, Turkey: Ministry of Health (Turkey), 2008.
Ministry of Health (Turkey). Turkey Health Statistics Yearbook 2014. Ankara, Turkey: General Directorate for Health Research (SAGEM) (Turkey), 2015.
Ministry of Health (Turkey). Turkey Health Statistics Yearbook 2015. Ankara, Turkey: General Directorate for Health Research (SAGEM) (Turkey), 2015.
Ministry of Health (Turkey). Turkey Health Statistics Yearbook 2016. Ankara, Turkey: General Directorate for Health Research (SAGEM) (Turkey), 2017.
Ministry of Health (Turkey). Turkey Health Statistics Yearbook 2017. Ankara, Turkey: Ministry of Health (Turkey), 2018.
Ministry of Health and Medical Education (Iran). Iran National Cancer Registry Report 2004-2005.
Ministry of Health and Population (Egypt), National Cancer Registry Program of Egypt. Egypt - Aswan National Cancer Registry Report 2008. Cairo, Egypt: National Cancer Registry Program of Egypt, 2010.
Ministry of Health and Population (Egypt), National Cancer Registry Program of Egypt. Egypt - Damietta National Cancer Registry Report 2009. Cairo, Egypt: National Cancer Registry Program of Egypt, 2011.
Ministry of Health of Turkey, Turkish Statistical Institute. Turkey Health Statistics Yearbook 2008. Ankara, Turkey
Ministry of Health of Turkey, Turkish Statistical Institute. Turkey Health Statistics Yearbook 2009. Ankara, Turkey
Ministry of Health of Turkey, Turkish Statistical Institute. Turkey Health Statistics Yearbook 2010. Ankara, Turkey
Ministry of Health, Population and Hospital Reform (Algeria), University Hospital of Batna. Algeria - Batna Cancer Registy Report 2000-2006. Batna, Algeria: University Hospital of Batna.
Ministry of Public Health (Lebanon), United Nations High Commissioner for Refugees (UNHCR), World Health Organization (WHO). Lebanon Statistical Bulletin 2013. Beirut, Lebanon: Ministry of Public Health (Lebanon).
Ministry of Public Health (Lebanon). Lebanon National Cancer Registry Tables 2005. Beirut, Lebanon: Ministry of Public Health (Lebanon).

Suggested citation
Ministry of Public Health (Lebanon). Lebanon National Cancer Registry Tables 2006. Beirut, Lebanon: Ministry of Public Health (Lebanon).
Ministry of Public Health (Lebanon). Lebanon National Cancer Registry Tables 2007. Beirut, Lebanon: Ministry of Public Health (Lebanon).
Ministry of Public Health (Lebanon). Lebanon Statistical Bulletin 2012. Beirut, Lebanon: Ministry of Public Health (Lebanon).
National Cancer Registry (Saudi Arabia). Saudi Arabia Cancer Incidence Report 1994-1996. Riyadh, Saudi Arabia: National Cancer Registry (Saudi Arabia), 1999.
National Cancer Registry (Saudi Arabia). Saudi Arabia Cancer Incidence Report 1997-1998. Riyadh, Saudi Arabia: National Cancer Registry (Saudi Arabia), 2001.
National Cancer Registry (Saudi Arabia). Saudi Arabia Cancer Incidence Report 1999-2000. Riyadh, Saudi Arabia: National Cancer Registry (Saudi Arabia), 2004.
National Cancer Registry (Saudi Arabia). Saudi Arabia Cancer Incidence Report 2001. Riyadh, Saudi Arabia: National Cancer Registry (Saudi Arabia), 2005.
National Cancer Registry (Saudi Arabia). Saudi Arabia Cancer Incidence Report 2002. Riyadh, Saudi Arabia: National Cancer Registry (Saudi Arabia).
National Cancer Registry (Saudi Arabia). Saudi Arabia Cancer Incidence Report 2003. Riyadh, Saudi Arabia: National Cancer Registry (Saudi Arabia).
National Cancer Registry Program of Egypt. Egypt - Aswan National Cancer Registry Statistics 2008. Cairo, Egypt: National Cancer Registry Program of Egypt.
National Cancer Registry Program of Egypt. Egypt - Aswan National Cancer Registry Statistics 2009. Cairo, Egypt: National Cancer Registry Program of Egypt.
National Cancer Registry Program of Egypt. Egypt - Aswan National Cancer Registry Statistics 2010. Cairo, Egypt: National Cancer Registry Program of Egypt.
National Cancer Registry Program of Egypt. Egypt - Damietta National Cancer Registry Statistics 2009. Cairo, Egypt: National Cancer Registry Program of Egypt.
National Cancer Registry Program of Egypt. Egypt - Damietta National Cancer Registry Statistics 2010. Cairo, Egypt: National Cancer Registry Program of Egypt.
National Cancer Registry Program of Egypt. Egypt - Damietta National Cancer Registry Statistics 2011. Cairo, Egypt: National Cancer Registry Program of Egypt.

Suggested citation
National Cancer Registry Program of Egypt. Egypt - Damietta National Cancer Registry Statistics 2012. Cairo, Egypt: National Cancer Registry Program of Egypt.
National Cancer Registry Program of Egypt. Egypt - El-Minia National Cancer Registry Statistics 2009. Cairo, Egypt: National Cancer Registry Program of Egypt.
National Health Authority (Qatar), Qatar Statistics Authority, World Health Organization (WHO). Qatar World Health Survey 2006.
National Institute of Public Health (Algeria). Algeria - Algiers Cancer Registry Report 2003. Alger, Algeria: National Institute of Public Health (Algeria).
National Institute of Public Health (Algeria). Algeria - Algiers Cancer Registry Report 2004. Alger, Algeria: National Institute of Public Health (Algeria).
National Institute of Public Health (Algeria). Algeria - Algiers Cancer Registry Report 2006. Alger, Algeria: National Institute of Public Health (Algeria).
National Institute of Public Health (Algeria). Algeria - Algiers Cancer Registry Report 2007. Alger, Algeria: National Institute of Public Health (Algeria).
Oran Cancer Registry (Algeria). Algeria - Oran Cancer Registry Report 2005. Oran, Algeria: Oran Cancer Registry (Algeria), 2006.
Oran Cancer Registry (Algeria). Algeria - Oran Cancer Registry Report 2006. Oran, Algeria: Oran Cancer Registry (Algeria), 2007.
Planning and Statistics Authority (PSA) (Qatar). Qatar Vital Statistics Annual Bulletin 2018. Planning and Statistics Authority (PSA) (Qatar), 2019.
Sadjadi A, Malekzadeh R, Derakhshan MH, Sepehr A, Nouraie M, Sotoudeh M, Yazdanbod A, Shokoohi B, Mashayekhi A, Arshi S, Majidpour A, Babaei M, Mosavi A, Mohagheghi MMA, Alimohammadian M. Cancer Occurrence in Ardabil: Results of a Population-Based Cancer Registry from Iran. Int J Cancer. 2003; 107: 113–118.
Saudi Cancer Registry. Saudi Arabia Cancer Incidence Report 2004. Riyadh, Saudi Arabia: Saudi Cancer Registry.
Saudi Cancer Registry. Saudi Arabia Cancer Incidence Report 2005. Riyadh, Saudi Arabia: Saudi Cancer Registry.
Saudi Cancer Registry. Saudi Arabia Cancer Incidence Report 2006. Riyadh, Saudi Arabia: Saudi Cancer Registry.
Saudi Cancer Registry. Saudi Arabia Cancer Incidence Report 2008. Riyadh, Saudi Arabia: Saudi Cancer Registry.

Suggested citation
Saudi Cancer Registry. Saudi Arabia Cancer Incidence Report 2009. Riyadh, Saudi Arabia: Saudi Cancer Registry, 2012.
Saudi Cancer Registry. Saudi Arabia Cancer Incidence Report 2010. Riyadh, Saudi Arabia: Saudi Cancer Registry, 2014.
Saudi Cancer Registry. Saudi Arabia Cancer Incidence Report 2011. Riyadh, Saudi Arabia: Saudi Cancer Registry, 2014.
Saudi Cancer Registry. Saudi Arabia Cancer Incidence Report 2012. Riyadh, Saudi Arabia: Saudi Cancer Registry, 2015.
Semnani S, Sadjadi A, Fahimi S, Nouraie M, Naeimi M, Kabir J, Fakheri H, Saadatnia H, Ghavamnasiri MR, Malekzadeh R. Declining incidence of esophageal cancer in the Turkmen Plain, eastern part of the Caspian Littoral of Iran: A retrospective cancer surveillance. Cancer Detect Prev. 2006; 30: 14-19.
Shamseddine A, Sibai A-M, Gehchan N, Rahal B, El-Saghir N, Ghosn M, Aftimos G, Chamsuddine N, Seoud M, Lebanese Cancer Epidemiology Group. Cancer Incidence in Postwar Lebanon: Findings from the First National Population-based Registry, 1998. Ann Epidemiol. 2004; 14(9): 663-8.
Thun MJ, Hannan LM, Adams-Campbell LL, Boffetta P, Buring JE, Feskanich D, Flanders WD, Jee SH, Katanoda K, Kolonel LN, Lee IM, Marugame T, Palmer JR, Riboli E, Sobue T, Avila-Tang E, Wilkens LR, Samet JM. Lung cancer occurrence in never-smokers: an analysis of 13 cohorts and 22 cancer registry studies. PLoS Med. 2008; 5(9):
Turkish Statistical Institute. Turkey Statistical Yearbook 2010. Ankara, Turkey: Turkish Statistical Institute, 2011.
Turkish Statistical Institute. Turkey Statistical Yearbook 2011. Ankara, Turkey: Turkish Statistical Institute, 2012.

Supplementary table 2

Location		Sex	New cases		Expected new cases in 2019		% 1990 - 2019 new cases change cause			% 1990 - 2019 new cases overall change
			1990	2019	Population growth	Population growth + Aging	Population growth	Age structure change	Incidence rate change	
North Africa and Middle East		Female	4,120	15,838	7,162	10,390	73.8%	78.4%	132.2%	284.4%
		Male	24,925	55,843	44,594	63,758	78.9%	76.9%	-31.8%	124.0%
Country	Afghanistan	Female	139	365	450	285	223.0%	-118.2%	57.1%	162.0%
		Male	809	1,111	2,811	1,251	247.7%	-193.0%	-17.2%	37.4%
	Algeria	Female	238	716	393	662	65.2%	113.0%	22.5%	200.7%
		Male	1,137	2,477	1,885	3,273	65.8%	122.1%	-70.0%	117.9%
	Bahrain	Female	12	37	29	56	155.2%	233.1%	-168.0%	220.3%
		Male	48	104	146	304	204.8%	328.2%	-416.7%	116.4%
	Egypt	Female	554	1,917	973	1,115	75.5%	25.6%	144.8%	245.9%
		Male	1,463	4,206	2,637	3,466	80.2%	56.7%	50.5%	187.4%
	Iran (Islamic Republic of)	Female	590	2,827	855	1,741	44.9%	150.3%	184.0%	379.2%
		Male	2,275	5,878	3,256	6,119	43.1%	125.8%	-10.6%	158.3%
	Iraq	Female	205	1,157	489	622	138.4%	64.5%	261.1%	464.0%
		Male	1,013	2,997	2,435	3,037	140.3%	59.4%	-4.0%	195.7%
	Jordan	Female	29	218	88	141	201.6%	180.6%	263.3%	645.5%
		Male	138	697	434	713	214.6%	202.1%	-11.8%	405.0%
	Kuwait	Female	15	53	42	74	173.6%	211.7%	-141.3%	243.9%
		Male	54	173	127	224	135.2%	180.2%	-95.5%	220.0%
	Lebanon	Female	98	507	159	241	62.2%	84.1%	271.3%	417.7%
		Male	384	914	592	789	54.0%	51.4%	32.5%	137.9%
	Libya	Female	27	128	43	78	61.1%	128.5%	187.3%	376.9%
		Male	339	797	532	885	57.0%	104.4%	-26.1%	135.4%
	Morocco	Female	170	656	240	392	40.9%	89.0%	154.9%	284.7%
		Male	1,885	4,622	2,703	4,414	43.4%	90.8%	11.0%	145.2%
	Oman	Female	11	45	22	27	101.3%	49.5%	169.2%	320.0%
		Male	48	102	126	140	160.6%	29.6%	-79.9%	110.3%
	Palestine	Female	26	113	62	69	136.7%	27.8%	169.5%	334.0%
		Male	147	410	356	428	142.1%	49.0%	-12.2%	179.0%
	Qatar	Female	2	21	11	15	392.2%	194.9%	308.1%	895.2%
		Male	15	103	104	156	618.7%	355.2%	-363.3%	610.6%

Location	Sex	New cases		Expected new cases in 2019		% 1990 - 2019 new cases change cause			% 1990 - 2019 new cases overall change
		1990	2019	Population growth	Population growth + Aging	Population growth	Age structure change	Incidence rate change	
Saudi Arabia	Female	68	429	144	210	110.2%	96.8%	322.0%	529.0%
	Male	353	1,115	822	1,098	132.5%	78.3%	4.9%	215.7%
Sudan	Female	117	403	235	234	101.3%	-0.5%	144.6%	245.4%
	Male	599	1,121	1,214	1,225	102.8%	1.8%	-17.2%	87.3%
Syrian Arab Republic	Female	117	382	138	275	18.0%	116.9%	90.5%	225.5%
	Male	427	991	456	984	7.0%	123.8%	1.5%	132.2%
Tunisia	Female	62	266	87	165	39.6%	127.1%	162.2%	328.9%
	Male	900	2,196	1,212	2,143	34.7%	103.4%	5.9%	144.0%
Turkey	Female	1,559	5,185	2,130	3,861	36.6%	111.0%	84.9%	232.5%
	Male	12,424	24,326	16,848	30,229	35.6%	107.7%	-47.5%	95.8%
United Arab Emirates	Female	12	108	44	85	282.9%	347.2%	199.1%	829.2%
	Male	50	434	280	519	454.1%	474.4%	-169%	759.5%
Yemen	Female	65	289	151	177	131.2%	40.2%	170.1%	341.5%
	Male	401	1,014	914	1,117	127.7%	50.5%	-25.7%	152.6%

Supplementary table 3

Country	Measure	Age-standardized rate (per 100,000)						% Change (1990 to 2019)		
		1990			2019					
		Both	Female	Male	Both	Female	Male	Both	Female	Male
Afghanistan	Incidence	13.2 (6.91 to 22.44)	3.96 (2.85 to 6.96)	21.48 (10.05 to 38.47)	11.74 (7.33 to 18.53)	5.1 (3.62 to 7.06)	19.22 (11.28 to 33.01)	-11.1 (-37.4 to 33.6)	28.6 (-17.9 to 98.1)	-10.5 (-37.9 to 43.3)
	Prevalence	12.41 (6.16 to 21.21)	3.84 (2.74 to 6.9)	20.23 (8.94 to 36.9)	11.25 (6.78 to 17.88)	5.05 (3.43 to 7.12)	18.26 (10.39 to 31.65)	-9.4 (-36.8 to 37.8)	31.5 (-15.9 to 102.8)	-9.7 (-37.8 to 47.3)
	Deaths	14.07 (7.61 to 23.87)	4.14 (2.95 to 7.17)	22.93 (11.06 to 40.54)	12.53 (7.99 to 19.7)	5.33 (3.89 to 7.3)	20.59 (12.25 to 34.99)	-11 (-37 to 34)	28.8 (-16.8 to 98.2)	-10.2 (-37.1 to 42.6)
	DALYs	339.29 (166.83 to 577.65)	109.55 (76.86 to 199.49)	550.37 (239.23 to 1005.39)	302.57 (179.92 to 481.25)	139.69 (92.77 to 198.61)	485.96 (274.05 to 844.56)	-10.8 (-37.7 to 35.9)	27.5 (-19 to 96)	-11.7 (-39.4 to 45.1)
	YLLs	336.32 (165.54 to 572.87)	108.55 (76.06 to 197.91)	545.61 (237.47 to 995.41)	299.95 (177.99 to 477.2)	138.46 (91.83 to 197.1)	481.77 (271.31 to 837.81)	-10.8 (-37.7 to 35.7)	27.5 (-19 to 96)	-11.7 (-39.4 to 45.4)
	YLDs	2.97 (1.33 to 5.66)	0.99 (0.6 to 1.75)	4.76 (1.85 to 9.66)	2.62 (1.44 to 4.41)	1.23 (0.74 to 1.91)	4.19 (2.08 to 7.74)	-11.8 (-39.8 to 39.1)	24.1 (-23.6 to 93.2)	-12 (-41.8 to 54.1)
Algeria	Incidence	11.76 (9.57 to 14.55)	4 (3.23 to 4.97)	19.41 (15.63 to 24.53)	9.79 (7.6 to 12.52)	4.46 (3.56 to 5.45)	14.83 (11.11 to 19.79)	-16.7 (-40 to 14.4)	11.4 (-19.3 to 48.8)	-23.6 (-47 to 9.3)
	Prevalence	11.21 (9.06 to 14)	3.92 (3.12 to 4.89)	18.61 (14.84 to 23.49)	9.75 (7.51 to 12.52)	4.76 (3.76 to 5.9)	14.52 (10.78 to 19.43)	-13 (-37.8 to 20.9)	21.4 (-12.2 to 62.9)	-22 (-46.2 to 11)
	Deaths	12.75 (10.41 to 15.73)	4.27 (3.46 to 5.3)	20.95 (16.89 to 26.46)	10.46 (8.16 to 13.36)	4.61 (3.69 to 5.63)	15.93 (11.99 to 21.17)	-18 (-40.2 to 11.6)	7.9 (-21.6 to 42.3)	-24 (-46.7 to 8)
	DALYs	286.19 (230.72 to 356.41)	99.89 (79.49 to 124.64)	476.29 (378.4 to 602.39)	231.8 (177.95 to 298.75)	102.76 (80.69 to 127.54)	355.62 (262.98 to 474.03)	-19 (-42.6 to 13.1)	2.9 (-25.8 to 38.8)	-25.3 (-48.7 to 6.4)
	YLLs	283.52 (229.03 to 353.69)	98.93 (78.76 to 123.23)	471.91 (375.08 to 597.88)	229.55 (176.21 to 295.95)	101.69 (79.85 to 126.04)	352.25 (261.13 to 470.38)	-19 (-42.6 to 13.1)	2.8 (-25.8 to 38.5)	-25.4 (-48.6 to 6.4)
	YLDs	2.67 (1.71 to 3.78)	0.95 (0.61 to 1.38)	4.38 (2.76 to 6.27)	2.25 (1.45 to 3.25)	1.07 (0.73 to 1.53)	3.36 (2.07 to 5.04)	-15.8 (-41.3 to 22.8)	12.4 (-23.9 to 59.1)	-23.3 (-50.1 to 17.2)

Country	Measure	Age-standardized rate (per 100,000)						% Change (1990 to 2019)		
		1990			2019					
		Both	Female	Male	Both	Female	Male	Both	Female	Male
Bahrain	Incidence	41.42 (35.26 to 47.78)	17.29 (14.65 to 20.25)	65 (54.39 to 75.63)	19.84 (15.2 to 25.09)	12.46 (9.96 to 15.05)	26.5 (19.94 to 34.33)	-52.1 (-64.7 to -34.8)	-27.9 (-44.8 to -7.5)	-59.2 (-71.3 to -41.6)
	Prevalence	36.04 (30.59 to 41.69)	15.54 (13.24 to 18.21)	55.12 (46.31 to 64.4)	18.01 (13.69 to 22.9)	12.83 (10.19 to 15.62)	22.56 (16.86 to 29.66)	-50 (-63.6 to -31.7)	-17.4 (-37.9 to 6.5)	-59.1 (-71.1 to -40.8)
	Deaths	46.06 (39.19 to 53.05)	18.98 (16.09 to 22.24)	73.32 (61.39 to 85.18)	22.08 (17.07 to 27.74)	13.04 (10.45 to 15.76)	30.42 (22.96 to 39.13)	-52.1 (-64.5 to -34.7)	-31.3 (-47.3 to -11.7)	-58.5 (-70.8 to -40.7)
	DALYs	903.87 (766.19 to 1045.64)	376.71 (319.16 to 442.35)	1387.81 (1164.27 to 1626.09)	392.08 (294.96 to 503.78)	238.46 (189.98 to 289.87)	518.94 (386.88 to 684.76)	-56.6 (-68.3 to -40.2)	-36.7 (-52.1 to -18.3)	-62.6 (-73.7 to -45.7)
	YLLs	895.26 (759.48 to 1036.04)	373.07 (316.45 to 437.83)	1374.43 (1151.64 to 1609.48)	387.93 (291.97 to 498.44)	235.76 (188 to 286.43)	513.51 (382.44 to 678)	-56.7 (-68.4 to -40.2)	-36.8 (-52.2 to -18.4)	-62.6 (-73.7 to -45.8)
	YLDs	8.61 (5.9 to 11.32)	3.64 (2.4 to 4.95)	13.38 (9.12 to 17.76)	4.15 (2.76 to 5.86)	2.7 (1.82 to 3.79)	5.43 (3.52 to 7.91)	-51.8 (-66.1 to -30.9)	-25.7 (-50 to 5.2)	-59.4 (-72.9 to -39.6)
Egypt	Incidence	6.57 (5.96 to 7.19)	3.69 (3.38 to 4.01)	9.44 (8.33 to 10.53)	9.22 (6.48 to 12.46)	6.61 (4.17 to 9.41)	11.51 (8.03 to 16.11)	40.2 (-4.5 to 92.4)	79.4 (13.3 to 159.5)	21.8 (-18.5 to 72.8)
	Prevalence	6.62 (6.01 to 7.27)	3.67 (3.35 to 4)	9.54 (8.4 to 10.69)	9.46 (6.65 to 12.78)	6.86 (4.36 to 9.82)	11.76 (8.16 to 16.52)	42.9 (-3.5 to 96.3)	87.2 (19.1 to 172.8)	23.4 (-17.6 to 75.3)
	Deaths	6.82 (6.18 to 7.48)	3.86 (3.54 to 4.21)	9.78 (8.61 to 10.87)	9.5 (6.68 to 12.9)	6.82 (4.3 to 9.71)	11.85 (8.3 to 16.67)	39.3 (-4.9 to 91.5)	76.7 (11.2 to 155.2)	21.2 (-18.7 to 71.6)
	DALYs	178.37 (162.3 to 195.72)	97.57 (89.08 to 106.12)	258.13 (228.51 to 288.75)	240.85 (169.37 to 326.92)	162.36 (103.36 to 233.19)	310.42 (213.22 to 437.29)	35 (-8.4 to 85.7)	66.4 (6.2 to 139.4)	20.3 (-20.2 to 71.9)
	YLLs	176.8 (161.07 to 193.81)	96.64 (88.36 to 105.05)	255.92 (226.95 to 286.67)	238.69 (167.94 to 324.35)	160.82 (102.1 to 230.94)	307.72 (211.64 to 433.74)	35 (-8.5 to 85.6)	66.4 (6.3 to 139.4)	20.2 (-20.3 to 71.7)
	YLDs	1.57 (1.05 to 2.11)	0.92 (0.64 to 1.23)	2.21 (1.45 to 3.12)	2.16 (1.25 to 3.31)	1.54 (0.84 to 2.46)	2.71 (1.49 to 4.34)	37.5 (-13.4 to 100.5)	67.1 (-2.1 to 149.8)	22.2 (-27.7 to 94)

Country	Measure	Age-standardized rate (per 100,000)						% Change (1990 to 2019)		
		1990			2019					
		Both	Female	Male	Both	Female	Male	Both	Female	Male
Iran (Islamic Republic of)	Incidence	11.07 (9.1 to 13.35)	4.74 (3.98 to 6.57)	17.18 (13.81 to 20.86)	12.24 (11.26 to 13.16)	7.94 (7.13 to 8.68)	16.58 (15.1 to 18.09)	10.6 (-13.1 to 41.9)	67.4 (10.9 to 108.5)	-3.5 (-25.2 to 28)
	Prevalence	10.76 (8.93 to 12.93)	4.76 (4.05 to 6.59)	16.38 (13.27 to 19.85)	12.53 (11.58 to 13.47)	8.84 (7.96 to 9.68)	16.26 (14.83 to 17.72)	16.4 (-8.2 to 47.9)	85.8 (21.5 to 129.2)	-0.7 (-23.3 to 31.5)
	Deaths	11.83 (9.73 to 14.37)	5.05 (4.2 to 7.05)	18.52 (14.8 to 22.63)	12.88 (11.86 to 13.89)	8.03 (7.19 to 8.8)	17.78 (16.23 to 19.41)	8.9 (-14.4 to 40.8)	59 (3.7 to 99.2)	-4 (-26.2 to 27.9)
	DALYs	275.77 (230.92 to 333.38)	120.14 (102.48 to 167.9)	420.75 (340.33 to 512.02)	286.82 (266.09 to 307.6)	178.15 (161.41 to 192.43)	396.94 (364.05 to 434.82)	4 (-18.2 to 32.5)	48.3 (-3.3 to 80.9)	-5.7 (-27.7 to 24.6)
	YLLs	273.27 (228.84 to 330.65)	119.01 (101.58 to 166.37)	416.93 (337.5 to 508.15)	284.04 (263.26 to 304.47)	176.3 (159.61 to 190.48)	393.22 (360.8 to 430.72)	3.9 (-18.2 to 32.4)	48.1 (-3.4 to 80.8)	-5.7 (-27.8 to 24.6)
	YLDs	2.5 (1.72 to 3.4)	1.13 (0.75 to 1.63)	3.82 (2.6 to 5.27)	2.78 (1.99 to 3.59)	1.86 (1.32 to 2.42)	3.72 (2.65 to 4.84)	11 (-13 to 40.5)	65 (9 to 105.9)	-2.7 (-25.5 to 28.6)
Iraq	Incidence	15.89 (12.7 to 19.67)	5.1 (3.59 to 8.41)	27.34 (21.24 to 34.5)	18.69 (14.53 to 22.68)	9.79 (7.53 to 12.32)	28.37 (21.98 to 34.02)	17.6 (-11.4 to 57.6)	92.2 (4.2 to 202.7)	3.8 (-23.9 to 40.7)
	Prevalence	15.37 (12.18 to 18.98)	5.16 (3.64 to 8.57)	26.07 (20.03 to 32.98)	18.76 (14.44 to 23.11)	10.7 (8.13 to 13.75)	27.36 (20.93 to 33.27)	22.1 (-10 to 65.5)	107.6 (12.2 to 229.8)	5 (-25 to 44.2)
	Deaths	16.78 (13.46 to 20.75)	5.25 (3.69 to 8.6)	29.12 (22.71 to 36.67)	19.86 (15.62 to 23.82)	9.9 (7.7 to 12.23)	30.87 (24.16 to 36.78)	18.3 (-10.3 to 57.2)	88.5 (2.5 to 191.7)	6 (-21 to 42.2)
	DALYs	403.58 (319.62 to 500.11)	134.65 (95.25 to 223.74)	683.37 (523.87 to 865.39)	451.59 (346.04 to 557.52)	239.59 (181.03 to 307.77)	675.57 (516.66 to 825.13)	11.9 (-18.2 to 52)	77.9 (-3.5 to 181.1)	-1.1 (-29.5 to 35.5)
	YLLs	400.03 (316.57 to 495.57)	133.43 (94.57 to 221.36)	677.38 (518.72 to 858.39)	447.42 (343.55 to 552.85)	237.32 (179.25 to 305.48)	669.35 (511.66 to 817.7)	11.8 (-18.3 to 52.1)	77.9 (-3.5 to 180.9)	-1.2 (-29.6 to 35.5)
	YLDs	3.55 (2.3 to 4.97)	1.23 (0.72 to 2.08)	5.99 (3.81 to 8.6)	4.17 (2.74 to 5.92)	2.27 (1.44 to 3.38)	6.22 (4 to 8.91)	17.5 (-17.6 to 66.6)	85.1 (-3.1 to 217.2)	3.7 (-30.7 to 50.3)

Country	Measure	Age-standardized rate (per 100,000)						% Change (1990 to 2019)		
		1990			2019					
		Both	Female	Male	Both	Female	Male	Both	Female	Male
Jordan	Incidence	12.49 (10.18 to 15.37)	4.46 (3.5 to 6.19)	20.39 (16.18 to 25.39)	14.34 (11.86 to 17.36)	7.19 (5.69 to 9.03)	20.9 (16.3 to 26.2)	14.8 (-13.2 to 52.5)	61.2 (7.8 to 130.3)	2.5 (-26.9 to 42.6)
	Prevalence	12.33 (10.03 to 15.14)	4.42 (3.47 to 6.03)	19.93 (15.83 to 24.92)	14.58 (12.05 to 17.57)	8.08 (6.35 to 10.25)	20.51 (15.91 to 25.81)	18.2 (-11.2 to 57.9)	82.6 (23.4 to 162.5)	2.9 (-27.5 to 45.6)
	Deaths	13.22 (10.79 to 16.31)	4.69 (3.71 to 6.31)	21.69 (17.24 to 27.13)	15.11 (12.47 to 18.25)	7.21 (5.7 to 9.09)	22.35 (17.53 to 27.92)	14.3 (-13.2 to 51)	53.9 (3.5 to 121.5)	3 (-25.8 to 42.7)
	DALYs	316.65 (257.4 to 393.73)	111.75 (88.36 to 149.03)	513.07 (408.98 to 643.19)	338.02 (276.61 to 409.77)	161.92 (128.25 to 205.65)	497.83 (385.83 to 628.29)	6.7 (-20.2 to 43.8)	44.9 (-3.2 to 106)	-3 (-31.4 to 36.9)
	YLLs	313.8 (255.71 to 389.97)	110.69 (87.23 to 147.59)	508.48 (404.14 to 638.14)	334.75 (273.97 to 405.67)	160.22 (126.86 to 203.8)	493.14 (382.41 to 622.33)	6.7 (-20.2 to 43.8)	44.7 (-3.5 to 106)	-3 (-31.4 to 37)
	YLDs	2.85 (1.94 to 3.99)	1.06 (0.68 to 1.6)	4.59 (3.09 to 6.41)	3.26 (2.17 to 4.61)	1.7 (1.1 to 2.47)	4.69 (2.94 to 6.77)	14.5 (-18.5 to 60.7)	60 (2.3 to 142.3)	2.2 (-31.3 to 52.3)
Kuwait	Incidence	11.95 (10.68 to 13.41)	6.69 (5.43 to 8.48)	15.45 (13.69 to 17.78)	10.1 (8.19 to 12.19)	5.18 (4.08 to 6.47)	13.28 (10.29 to 16.6)	-15.5 (-30.8 to 4.2)	-22.5 (-44.8 to 8.6)	-14 (-32 to 8.5)
	Prevalence	12.08 (10.81 to 13.5)	7.22 (5.85 to 9.14)	15.13 (13.4 to 17.38)	10.32 (8.46 to 12.42)	6.63 (5.22 to 8.49)	12.72 (9.79 to 15.96)	-14.5 (-29.6 to 5.4)	-8.1 (-35.2 to 30.4)	-15.9 (-34 to 6.5)
	Deaths	12.5 (11.15 to 14.07)	6.76 (5.47 to 8.57)	16.46 (14.54 to 19.02)	10.75 (8.72 to 12.98)	4.96 (3.95 to 6.14)	14.51 (11.28 to 18.13)	-14 (-29.7 to 5.9)	-26.7 (-47.4 to 1.3)	-11.8 (-30.2 to 11.9)
	DALYs	286.2 (257.15 to 319.9)	156.73 (127.51 to 196.86)	366.03 (325.15 to 419.77)	214.8 (174.68 to 260.68)	104.23 (82.26 to 130.24)	287.61 (222.72 to 360.28)	-24.9 (-38.3 to -7.3)	-33.5 (-53.1 to -6.2)	-21.4 (-38.6 to -0.2)
	YLLs	283.45 (254.52 to 317.08)	155.15 (126.29 to 194.78)	362.52 (321.19 to 416.48)	212.52 (173.05 to 258.24)	102.98 (81.23 to 128.81)	284.66 (220.25 to 354.87)	-25 (-38.3 to -7.4)	-33.6 (-53.3 to -6.4)	-21.5 (-38.6 to -0.5)
	YLDs	2.75 (1.88 to 3.71)	1.58 (0.97 to 2.35)	3.51 (2.36 to 4.83)	2.28 (1.52 to 3.22)	1.25 (0.78 to 1.86)	2.95 (1.89 to 4.28)	-17.1 (-39.3 to 11.2)	-21 (-51 to 25)	-16 (-41.8 to 19.4)

Country	Measure	Age-standardized rate (per 100,000)						% Change (1990 to 2019)		
		1990			2019					
		Both	Female	Male	Both	Female	Male	Both	Female	Male
Lebanon	Incidence	20.94 (15.75 to 26.64)	8.46 (6.95 to 10.48)	33.75 (23.76 to 43.9)	27.29 (22.42 to 35.83)	17.75 (13.08 to 24.52)	38.92 (30.94 to 51.44)	30.3 (-6.5 to 97.9)	109.8 (28.7 to 191.7)	15.3 (-19.4 to 89.3)
	Prevalence	20.61 (15.41 to 26.17)	8.69 (7.02 to 10.79)	32.63 (22.71 to 42.99)	30.4 (24.38 to 39.49)	23.13 (16.79 to 31.66)	39.26 (30.94 to 51.72)	47.5 (4.1 to 125.5)	166.2 (64 to 273.3)	20.3 (-16.1 to 99.1)
	Deaths	22.08 (16.72 to 27.88)	8.75 (7.21 to 10.81)	35.93 (25.6 to 46.44)	27.51 (22.67 to 36.43)	16.51 (12.14 to 22.82)	40.95 (32.73 to 54.66)	24.6 (-10.1 to 89.9)	88.7 (14.9 to 161.6)	14 (-19.8 to 84.3)
	DALYs	529.92 (393.94 to 675.4)	215.2 (174.85 to 266.07)	847.46 (586.87 to 1117.95)	629.57 (507.29 to 817.1)	384.97 (281.37 to 520.49)	926 (725.13 to 1215.38)	18.8 (-15.4 to 78.6)	78.9 (12.1 to 150.2)	9.3 (-23.7 to 79.1)
	YLLs	525.27 (390.26 to 669.14)	213.25 (172.98 to 263.94)	840.06 (581.13 to 1106.23)	623.43 (501.81 to 809.15)	380.76 (278.3 to 514.12)	917.49 (716.97 to 1204.81)	18.7 (-15.5 to 78.4)	78.6 (12 to 149.9)	9.2 (-23.7 to 79.3)
	YLDs	4.65 (2.96 to 6.68)	1.96 (1.27 to 2.9)	7.39 (4.38 to 11.04)	6.15 (4.11 to 8.88)	4.21 (2.65 to 6.27)	8.51 (5.56 to 12.68)	32.2 (-10.4 to 103.1)	114.9 (23.4 to 221.9)	15.1 (-26 to 91.2)
Libya	Incidence	19.87 (15.19 to 25.29)	3.09 (2.24 to 4.97)	34.93 (26.49 to 45.3)	18.49 (13.72 to 23.47)	5.1 (3.19 to 6.89)	31.41 (22.96 to 40.16)	-7 (-36.6 to 35.5)	65 (-33 to 172.9)	-10.1 (-39.4 to 33.9)
	Prevalence	19.61 (15 to 25.08)	3.13 (2.29 to 5.08)	34.11 (25.85 to 44.51)	18.43 (13.68 to 23.46)	5.53 (3.52 to 7.56)	30.87 (22.55 to 39.69)	-6 (-36.5 to 38.6)	76.6 (-28.8 to 196.6)	-9.5 (-39.4 to 35.7)
	Deaths	20.95 (15.98 to 26.6)	3.21 (2.32 to 5.13)	37.04 (28.16 to 47.85)	19.4 (14.44 to 24.52)	5.13 (3.23 to 6.86)	33.22 (24.33 to 42.53)	-7.4 (-36.1 to 34.3)	59.8 (-34.7 to 163.4)	-10.3 (-39.6 to 32)
	DALYs	502.23 (383.81 to 643.87)	77.92 (56.98 to 127.01)	872.16 (659.4 to 1139)	457.78 (341.26 to 583.95)	123.7 (78.79 to 170.07)	778.79 (569.1 to 1000.8)	-8.9 (-38.9 to 34.6)	58.8 (-36.3 to 167.4)	-10.7 (-40.5 to 34.5)
	YLLs	497.79 (380.19 to 638.87)	77.14 (56.45 to 125.4)	864.45 (652.4 to 1128.76)	453.67 (338.39 to 579.04)	122.49 (77.89 to 168.16)	771.88 (565 to 992.62)	-8.9 (-38.9 to 34.5)	58.8 (-36.3 to 167.3)	-10.7 (-40.4 to 34.5)
	YLDs	4.45 (2.93 to 6.31)	0.78 (0.48 to 1.33)	7.71 (5.03 to 10.98)	4.11 (2.67 to 5.94)	1.2 (0.69 to 1.89)	6.91 (4.4 to 10.01)	-7.6 (-39.6 to 36.8)	54.4 (-35.7 to 163.1)	-10.3 (-44.7 to 34.9)

Country	Measure	Age-standardized rate (per 100,000)						% Change (1990 to 2019)		
		1990			2019					
		Both	Female	Male	Both	Female	Male	Both	Female	Male
Morocco	Incidence	14.6 (11.04 to 17.9)	2.48 (1.94 to 3.14)	26.97 (20.13 to 33.81)	16.36 (11.74 to 20.97)	4.17 (3.04 to 5.29)	28.7 (20.43 to 37.42)	12 (-20.2 to 50.7)	68.2 (11.1 to 139.3)	6.4 (-24.7 to 45.7)
	Prevalence	14.73 (11.23 to 18.12)	2.4 (1.87 to 3.03)	27.29 (20.5 to 34.18)	16.63 (11.96 to 21.41)	4.29 (3.11 to 5.53)	29.03 (20.68 to 37.91)	12.8 (-20.9 to 54.2)	78.8 (17.8 to 156.5)	6.4 (-25.5 to 47.6)
	Deaths	14.86 (11.22 to 18.24)	2.63 (2.06 to 3.33)	27.4 (20.3 to 34.18)	16.98 (12.17 to 21.74)	4.34 (3.16 to 5.52)	29.85 (21.29 to 38.84)	14.3 (-17.9 to 53.3)	65.1 (9.6 to 131.6)	8.9 (-23 to 49.7)
	DALYs	379.62 (289.93 to 468.08)	63.19 (49.76 to 79.81)	701.62 (526.97 to 875.36)	418.92 (302.65 to 542.15)	102.75 (74.49 to 132.83)	736.81 (528.3 to 963.86)	10.4 (-22.4 to 51.4)	62.6 (7.1 to 135.2)	5 (-26.4 to 46.6)
	YLLs	376.27 (287.4 to 462.99)	62.57 (49.27 to 79.05)	695.49 (523.45 to 867.85)	415.21 (299.97 to 537.12)	101.74 (73.85 to 131.61)	730.37 (523.34 to 953.73)	10.3 (-22.4 to 51.5)	62.6 (7 to 135.4)	5 (-26.5 to 46.7)
	YLDs	3.35 (2.16 to 4.71)	0.62 (0.41 to 0.9)	6.13 (3.9 to 8.75)	3.71 (2.33 to 5.46)	1.01 (0.64 to 1.47)	6.44 (3.94 to 9.54)	10.9 (-27.3 to 58.7)	63.5 (6.7 to 136.9)	5 (-31.9 to 54.4)
Oman	Incidence	9.21 (6.77 to 11.74)	3.72 (2.44 to 6.04)	14.7 (10.74 to 18.57)	9.86 (8.16 to 11.94)	6.72 (5.08 to 8.06)	12.86 (10.25 to 16.55)	7 (-24.7 to 53)	80.6 (-12 to 189.2)	-12.5 (-38.3 to 31.7)
	Prevalence	8.89 (6.48 to 11.48)	3.72 (2.43 to 6.03)	13.66 (9.93 to 17.5)	9.74 (7.92 to 12.01)	7.77 (5.74 to 9.33)	11.68 (9.06 to 15.5)	9.6 (-24.2 to 58.6)	108.9 (0.6 to 233.8)	-14.5 (-40.9 to 31.3)
	Deaths	9.74 (7.17 to 12.34)	3.88 (2.55 to 6.29)	15.9 (11.72 to 19.99)	10.45 (8.67 to 12.59)	6.6 (4.98 to 7.96)	14.17 (11.41 to 18.04)	7.2 (-24.5 to 52.4)	70.3 (-17 to 171.7)	-10.9 (-36.5 to 32.1)
	DALYs	226.09 (164.58 to 292.25)	90.69 (59.43 to 147.52)	348.22 (252.79 to 446.99)	211.73 (170.66 to 267.77)	141.83 (104.09 to 169.59)	274.3 (211.84 to 366.38)	-6.4 (-34.7 to 35.8)	56.4 (-24.1 to 147.6)	-21.2 (-45.7 to 21.4)
	YLLs	223.99 (163.04 to 289.7)	89.79 (58.84 to 146.23)	344.96 (251.16 to 442.04)	209.51 (168.35 to 265.06)	140.24 (103.03 to 167.77)	271.5 (209.46 to 363.03)	-6.5 (-34.8 to 35.6)	56.2 (-24.2 to 147.4)	-21.3 (-45.7 to 21)
	YLDs	2.1 (1.3 to 3.12)	0.9 (0.5 to 1.53)	3.26 (2.04 to 4.87)	2.21 (1.46 to 3.15)	1.59 (1 to 2.29)	2.8 (1.78 to 4.19)	5.3 (-29.8 to 61.1)	76.6 (-15.7 to 208.1)	-14 (-46.5 to 41.3)

Country	Measure	Age-standardized rate (per 100,000)						% Change (1990 to 2019)		
		1990			2019					
		Both	Female	Male	Both	Female	Male	Both	Female	Male
Palestine	Incidence	20.21 (15.26 to 26.61)	5.36 (3.67 to 8.3)	38.22 (28.79 to 49.99)	22.53 (19.09 to 26.1)	9.15 (7.46 to 10.99)	38.02 (32.52 to 43.98)	11.5 (-20.6 to 54.6)	70.6 (3 to 175.8)	-0.5 (-29.5 to 37.7)
	Prevalence	19.45 (14.51 to 25.84)	5.52 (3.8 to 8.57)	36.37 (26.93 to 48.22)	22.43 (18.96 to 26.21)	9.88 (8.01 to 11.96)	36.28 (30.96 to 42.04)	15.3 (-18.6 to 61.5)	79.1 (7.8 to 191.4)	-0.2 (-30.4 to 39)
	Deaths	21.61 (16.39 to 28.34)	5.55 (3.79 to 8.49)	41.16 (31.31 to 53.87)	23.83 (20.24 to 27.6)	9.3 (7.6 to 11.15)	41.19 (35.31 to 47.72)	10.3 (-21.1 to 51.8)	67.4 (0.6 to 170.9)	0.1 (-28.4 to 38.3)
	DALYs	500.96 (372.96 to 664.89)	139.86 (95.99 to 215.67)	938.13 (693.12 to 1243.03)	546.36 (462.77 to 639.36)	219.65 (178.84 to 265.01)	901.55 (768.02 to 1046.3)	9.1 (-23.3 to 52.7)	57.1 (-4.1 to 154.2)	-3.9 (-33 to 33.7)
	YLLs	496.49 (369.9 to 658.87)	138.57 (95.26 to 213.4)	929.8 (687.41 to 1230.14)	541.4 (458.56 to 634.45)	217.53 (176.96 to 262.5)	893.38 (760.71 to 1035.03)	9 (-23.4 to 52.7)	57 (-4.2 to 154.3)	-3.9 (-33 to 33.7)
	YLDs	4.47 (2.83 to 6.6)	1.28 (0.73 to 2.13)	8.33 (5.22 to 12.44)	4.96 (3.4 to 6.72)	2.13 (1.43 to 3.03)	8.17 (5.49 to 11.12)	11 (-24 to 61.9)	65.9 (-4.2 to 192)	-1.9 (-34.3 to 45.5)
Qatar	Incidence	17.63 (13.48 to 22.88)	5.96 (4.17 to 8.54)	26.6 (19.15 to 34.97)	18.78 (14.38 to 23.88)	11.8 (9.21 to 14.72)	21.19 (15.97 to 27.58)	6.5 (-29.7 to 57.1)	98.1 (20.5 to 212.3)	-20.3 (-49 to 28.6)
	Prevalence	16.31 (12.37 to 21.29)	5.73 (4.1 to 8.13)	23.56 (16.96 to 31.26)	17.54 (13.23 to 22.69)	13.47 (10.35 to 16.86)	18.97 (14.08 to 25.29)	7.5 (-30.7 to 64.3)	135.1 (43.2 to 258.9)	-19.5 (-49.6 to 32.5)
	Deaths	19.02 (14.57 to 24.68)	6.34 (4.39 to 9.21)	29.39 (21.24 to 38.4)	20.74 (16 to 26.07)	11.8 (9.31 to 14.65)	23.78 (18.07 to 30.42)	9 (-27 to 58)	86.2 (11.4 to 199.3)	-19.1 (-46.8 to 28)
	DALYs	408.48 (308.7 to 530.86)	139.91 (100.79 to 196.7)	588.1 (422.81 to 778.46)	373.88 (279.21 to 491.27)	220.52 (170.71 to 275.17)	426.9 (315.74 to 572.97)	-8.5 (-42.1 to 42.3)	57.6 (-1.1 to 137.6)	-27.4 (-54.9 to 20.7)
	YLLs	404.67 (306.12 to 526.4)	138.55 (99.83 to 194.65)	582.49 (419.23 to 771.51)	369.93 (276.57 to 486.93)	217.89 (168.73 to 271.85)	422.48 (312.69 to 568.2)	-8.6 (-42.1 to 42)	57.3 (-1.4 to 136.9)	-27.5 (-55 to 20.6)
	YLDs	3.81 (2.43 to 5.38)	1.35 (0.78 to 2.2)	5.61 (3.47 to 8.05)	3.96 (2.61 to 5.78)	2.63 (1.7 to 3.77)	4.42 (2.84 to 6.54)	3.7 (-36.4 to 61.9)	94.4 (8.2 to 225.7)	-21.3 (-52.9 to 33.7)

Country	Measure	Age-standardized rate (per 100,000)						% Change (1990 to 2019)		
		1990			2019					
		Both	Female	Male	Both	Female	Male	Both	Female	Male
Saudi Arabia	Incidence	7.51 (5.61 to 9.68)	2.8 (1.92 to 4.58)	11.08 (8.06 to 14.43)	8.77 (6.98 to 10.62)	5.46 (4.16 to 6.88)	10.94 (8.54 to 13.69)	16.8 (-19.2 to 71.6)	95.2 (-0.5 to 216.9)	-1.2 (-33.1 to 54.8)
	Prevalence	7.21 (5.39 to 9.3)	2.79 (1.91 to 4.53)	10.42 (7.49 to 13.76)	9.01 (7.13 to 10.91)	6.53 (4.9 to 8.28)	10.62 (8.17 to 13.27)	25 (-14.7 to 82.3)	133.9 (20 to 281.7)	1.9 (-31.9 to 61.1)
	Deaths	8.88 (6.61 to 11.41)	3.48 (2.35 to 5.84)	13.07 (9.48 to 17.06)	9.14 (7.33 to 11.06)	5.31 (4.06 to 6.69)	11.67 (9.15 to 14.5)	2.9 (-29.8 to 51.3)	52.6 (-24.4 to 154.2)	-10.7 (-39.2 to 37.5)
	DALYs	205.79 (149.91 to 271.26)	85.5 (57.52 to 138.18)	292.33 (208.87 to 389.81)	208.01 (162.62 to 253.25)	127.19 (95.79 to 162.81)	260.49 (199.92 to 324.97)	1.1 (-32.1 to 51.8)	48.8 (-24.1 to 147.5)	-10.9 (-40.8 to 40)
	YLLs	204.05 (148.48 to 269.31)	84.79 (56.82 to 136.9)	289.82 (206.99 to 386.56)	205.99 (161.13 to 250.45)	125.86 (94.84 to 161.15)	258.01 (197.9 to 321.39)	1 (-32.2 to 51.4)	48.4 (-24.4 to 147)	-11 (-40.9 to 39.8)
	YLDs	1.74 (1.11 to 2.54)	0.7 (0.42 to 1.24)	2.5 (1.52 to 3.75)	2.02 (1.31 to 2.9)	1.32 (0.85 to 1.91)	2.48 (1.54 to 3.67)	16.3 (-22.5 to 83.3)	88.3 (-4.2 to 214.1)	-1.1 (-39 to 71.7)
Sudan	Incidence	7.71 (4.56 to 14.04)	2.58 (1.78 to 3.8)	12.34 (6.24 to 23.76)	8.28 (5.39 to 12.91)	4.46 (3.16 to 5.92)	11.42 (6.94 to 19.59)	7.4 (-25.9 to 67.5)	73 (0 to 176.3)	-7.5 (-38.8 to 55.6)
	Prevalence	7.36 (4.25 to 13.48)	2.52 (1.78 to 3.66)	11.69 (5.8 to 22.73)	8.03 (5.16 to 12.46)	4.54 (3.15 to 6.14)	10.91 (6.61 to 18.67)	9.2 (-27.1 to 75.7)	80.4 (2.4 to 189.7)	-6.7 (-39.7 to 61.3)
	Deaths	8.23 (4.91 to 14.95)	2.72 (1.84 to 4.03)	13.24 (6.8 to 25.41)	8.79 (5.86 to 13.7)	4.61 (3.33 to 6.12)	12.23 (7.52 to 20.93)	6.9 (-26 to 64.8)	69.7 (-0.3 to 171.4)	-7.6 (-38 to 52.4)
	DALYs	195.66 (111.73 to 358.22)	68.08 (48.09 to 98.22)	310.24 (152.78 to 599.77)	203.83 (131.72 to 318.09)	112.65 (79 to 155.09)	279.38 (168.36 to 479.82)	4.2 (-29.9 to 69.5)	65.5 (-4.9 to 166.9)	-9.9 (-42 to 57.1)
	YLLs	193.87 (110.54 to 355.77)	67.43 (47.71 to 97.23)	307.42 (151.39 to 594.81)	201.91 (130.66 to 314.81)	111.56 (78.19 to 154)	276.76 (167.26 to 474.88)	4.1 (-29.9 to 69.5)	65.4 (-5 to 166.9)	-10 (-42 to 57.1)
	YLDs	1.79 (0.86 to 3.52)	0.65 (0.38 to 1.05)	2.82 (1.18 to 5.94)	1.92 (1.12 to 3.28)	1.08 (0.66 to 1.6)	2.61 (1.39 to 4.87)	7.4 (-30 to 76.5)	67.5 (-6.2 to 174.8)	-7.3 (-42.8 to 68.1)

Country	Measure	Age-standardized rate (per 100,000)						% Change (1990 to 2019)		
		1990			2019					
		Both	Female	Male	Both	Female	Male	Both	Female	Male
Syrian Arab Republic	Incidence	10.09 (7.75 to 12.72)	4.41 (3.25 to 6.05)	15.21 (11.61 to 19.24)	11.09 (8.24 to 14.52)	6.61 (4.56 to 8.78)	15.37 (11.3 to 20.13)	9.9 (-25 to 60.4)	49.9 (-19.1 to 133.5)	1.1 (-30.8 to 52.1)
	Prevalence	10.05 (7.71 to 12.64)	4.51 (3.36 to 6.13)	15.06 (11.39 to 19.27)	11.37 (8.39 to 15.01)	7.14 (4.89 to 9.67)	15.43 (11.28 to 20.4)	13.2 (-23.3 to 67.2)	58.2 (-13.6 to 146.9)	2.4 (-30.9 to 56.5)
	Deaths	10.63 (8.18 to 13.32)	4.61 (3.38 to 6.34)	16.04 (12.23 to 20.22)	11.62 (8.7 to 15.09)	6.82 (4.76 to 8.95)	16.16 (11.92 to 21.03)	9.3 (-25.3 to 58.6)	47.8 (-21 to 130.7)	0.7 (-30.5 to 49.5)
	DALYs	264.02 (203 to 331.81)	118.11 (88.86 to 159.85)	396.61 (299.78 to 508.96)	274.37 (202.98 to 363.54)	155.27 (106.32 to 210.2)	389.3 (283.36 to 517.06)	3.9 (-29.7 to 53.3)	31.5 (-28.7 to 103.2)	-1.8 (-33.9 to 49.3)
	YLLs	261.66 (200.94 to 329.55)	117.04 (88.12 to 158.37)	393.08 (297.49 to 503.68)	271.8 (201.11 to 360.69)	153.71 (105.27 to 208.59)	385.76 (280.84 to 512.03)	3.9 (-29.9 to 53.1)	31.3 (-28.7 to 102.8)	-1.9 (-33.9 to 49.4)
	YLDs	2.36 (1.51 to 3.39)	1.07 (0.68 to 1.64)	3.52 (2.18 to 5.12)	2.57 (1.59 to 3.72)	1.56 (0.92 to 2.25)	3.54 (2.15 to 5.33)	9 (-26.3 to 71.1)	45.9 (-21.9 to 140.6)	0.4 (-34.7 to 58.5)
Tunisia	Incidence	19.13 (15.32 to 23.04)	2.54 (2.01 to 3.19)	34.72 (27.7 to 41.93)	19.37 (13.86 to 26.55)	4.12 (3 to 5.52)	35.54 (24.95 to 49.18)	1.3 (-33 to 49.5)	62.2 (5.4 to 139.6)	2.3 (-33.1 to 52.6)
	Prevalence	18.54 (14.88 to 22.33)	2.58 (2.05 to 3.22)	33.54 (26.68 to 40.58)	19.87 (14.07 to 27.4)	4.82 (3.48 to 6.48)	35.6 (24.7 to 49.69)	7.2 (-29 to 58.2)	86.9 (21.7 to 180.9)	6.2 (-31 to 58.2)
	Deaths	20.38 (16.35 to 24.59)	2.65 (2.08 to 3.32)	37.18 (29.72 to 44.73)	20.16 (14.44 to 27.58)	4.03 (2.94 to 5.36)	37.46 (26.5 to 51.5)	-1.1 (-33.7 to 46.2)	52.3 (-1.2 to 125.5)	0.7 (-33.2 to 50)
	DALYs	468.58 (376.74 to 565.35)	63.61 (50.52 to 79.37)	848.97 (676.97 to 1028.67)	471.77 (328.88 to 654.33)	94.06 (67.51 to 126.56)	865.24 (600.52 to 1209.83)	0.7 (-33.3 to 48.8)	47.9 (-3.6 to 122.1)	1.9 (-33.6 to 51.7)
	YLLs	464.33 (373.1 to 560.37)	62.97 (50.08 to 78.68)	841.32 (670.53 to 1019.48)	467.44 (325.73 to 648.42)	93.03 (66.8 to 125.05)	857.43 (592.63 to 1198.35)	0.7 (-33.3 to 48.8)	47.7 (-3.6 to 121.7)	1.9 (-33.7 to 51.9)
	YLDs	4.25 (2.79 to 5.93)	0.64 (0.41 to 0.93)	7.65 (5 to 10.75)	4.33 (2.59 to 6.57)	1.02 (0.63 to 1.57)	7.81 (4.64 to 11.98)	1.8 (-34.2 to 57.9)	59.8 (3.9 to 144.9)	2.1 (-35 to 59.7)

Country	Measure	Age-standardized rate (per 100,000)						% Change (1990 to 2019)		
		1990			2019					
		Both	Female	Male	Both	Female	Male	Both	Female	Male
Turkey	Incidence	37.91 (29.95 to 46.82)	8.23 (6.69 to 11.29)	70.29 (54.12 to 88.3)	33.08 (26.25 to 41.11)	11.13 (8.87 to 13.83)	57.7 (45.57 to 72.04)	-12.7 (-38.2 to 19.7)	35.2 (-6.2 to 84.4)	-17.9 (-42.5 to 15.5)
	Prevalence	38.27 (29.91 to 47.54)	8.33 (6.81 to 11.45)	70.36 (53.55 to 89)	34.95 (27.58 to 43.51)	13.07 (10.33 to 16.36)	59 (46.56 to 73.95)	-8.7 (-35.4 to 26.9)	56.9 (8.8 to 114.5)	-16.1 (-41.9 to 20)
	Deaths	39.25 (31.21 to 48.16)	8.58 (6.94 to 11.7)	73.04 (56.52 to 91.55)	33.77 (26.89 to 41.8)	10.9 (8.71 to 13.53)	59.69 (47.29 to 74.06)	-14 (-38.8 to 17.6)	27.1 (-11.2 to 74.3)	-18.3 (-42.6 to 14)
	DALYs	1009.45 (782.79 to 1260.22)	214.49 (175.34 to 292.75)	1857.11 (1408.16 to 2350.19)	814.58 (643.77 to 1017)	250.26 (198.42 to 311.87)	1431.86 (1132.21 to 1796.08)	-19.3 (-43.1 to 12.7)	16.7 (-19.2 to 59.7)	-22.9 (-46.8 to 10.4)
	YLLs	1000.93 (776.14 to 1249.51)	212.56 (173.52 to 289.81)	1841.45 (1392.9 to 2332.18)	807.12 (638.54 to 1008.22)	247.62 (196.42 to 308.72)	1419.05 (1120.92 to 1780.36)	-19.4 (-43.1 to 12.7)	16.5 (-19.3 to 59.3)	-22.9 (-46.9 to 10.3)
	YLDs	8.52 (5.68 to 11.9)	1.92 (1.22 to 2.91)	15.66 (10.14 to 22.48)	7.46 (5.05 to 10.51)	2.64 (1.7 to 3.85)	12.82 (8.71 to 18.3)	-12.5 (-39.4 to 25.1)	37.3 (-10.1 to 101.5)	-18.2 (-45 to 19.4)
United Arab Emirates	Incidence	19.16 (14.25 to 24.68)	10.03 (5.98 to 17.33)	25.88 (18.55 to 32.48)	18.62 (13.54 to 25.75)	12.79 (7.5 to 19.26)	21.52 (15.82 to 29.85)	-2.8 (-35.2 to 51.6)	27.5 (-54 to 95.6)	-16.8 (-45.5 to 39.8)
	Prevalence	17.08 (12.77 to 21.97)	9.23 (5.58 to 15.77)	22.73 (16.38 to 29.08)	16.87 (12.24 to 23.29)	12.51 (7.54 to 18.28)	19.03 (13.97 to 26.33)	-1.2 (-34.1 to 53.8)	35.5 (-50.2 to 110)	-16.3 (-45.7 to 42.7)
	Deaths	21.14 (15.68 to 27.35)	11 (6.51 to 19.19)	28.67 (20.58 to 35.78)	20.35 (14.71 to 28.22)	13.55 (7.93 to 20.63)	23.74 (17.44 to 32.88)	-3.7 (-35.5 to 49.9)	23.2 (-55.4 to 87)	-17.2 (-45.8 to 40.3)
	DALYs	427.66 (318.73 to 549.51)	225.49 (136.24 to 389.51)	571.77 (411.92 to 730.64)	406.46 (295.09 to 559.14)	274.87 (164.71 to 396.5)	468.7 (343.4 to 645.66)	-5 (-36.9 to 47.9)	21.9 (-54.8 to 88.7)	-18 (-46.8 to 39)
	YLLs	423.56 (316.2 to 544.03)	223.31 (135.09 to 385.85)	566.29 (408.67 to 722.68)	402.49 (292.42 to 552.97)	272.1 (163.12 to 391.86)	464.16 (340.23 to 640.34)	-5 (-37 to 47.9)	21.9 (-54.8 to 88.1)	-18 (-46.7 to 38.8)
	YLDs	4.09 (2.57 to 5.92)	2.19 (1.05 to 4.14)	5.48 (3.38 to 7.84)	3.96 (2.41 to 6.22)	2.77 (1.43 to 4.68)	4.54 (2.74 to 7.05)	-3.2 (-39.9 to 54.3)	26.7 (-50.5 to 115.8)	-17.1 (-50.1 to 46.4)

Country	Measure	Age-standardized rate (per 100,000)						% Change (1990 to 2019)		
		1990			2019					
		Both	Female	Male	Both	Female	Male	Both	Female	Male
Yemen	Incidence	9.4 (6.1 to 14.63)	2.57 (1.57 to 4.48)	17.42 (10.62 to 28.06)	9.95 (6.8 to 14.67)	4.15 (3.11 to 5.57)	15.94 (10.39 to 24.91)	5.8 (-25.9 to 59.4)	61.6 (-17.3 to 179.7)	-8.5 (-38.4 to 41)
	Prevalence	9.12 (5.85 to 14.37)	2.49 (1.55 to 4.28)	16.41 (9.86 to 26.73)	9.57 (6.56 to 14.22)	4.18 (3.08 to 5.65)	15.12 (9.8 to 23.75)	5 (-27.8 to 61.4)	67.9 (-14.9 to 195.1)	-7.8 (-39 to 45.1)
	Deaths	9.89 (6.42 to 15.39)	2.71 (1.64 to 4.79)	18.65 (11.47 to 29.87)	10.61 (7.26 to 15.61)	4.34 (3.25 to 5.81)	17.12 (11.19 to 26.67)	7.2 (-24.7 to 60.2)	59.9 (-18 to 178)	-8.2 (-37.7 to 39.9)
	DALYs	244.3 (155.48 to 383.05)	67.22 (42.18 to 114.81)	436.24 (261.48 to 717.04)	248.83 (169.31 to 368.91)	107.43 (78.67 to 145.39)	393.94 (254.61 to 622.7)	1.9 (-30.1 to 57.5)	59.8 (-18.9 to 182.9)	-9.7 (-40.2 to 43.8)
	YLLs	242.12 (153.96 to 380.16)	66.57 (41.73 to 113.64)	432.31 (259.15 to 710.04)	246.54 (167.95 to 365.21)	106.41 (77.99 to 144.12)	390.33 (252.32 to 616.52)	1.8 (-30.2 to 57.5)	59.8 (-18.8 to 182.9)	-9.7 (-40.4 to 43.9)
	YLDs	2.18 (1.2 to 3.73)	0.64 (0.34 to 1.19)	3.93 (1.99 to 6.98)	2.29 (1.35 to 3.77)	1.01 (0.66 to 1.5)	3.61 (2 to 6.12)	5.2 (-32.8 to 66.3)	57.3 (-20.2 to 178.1)	-8 (-44.8 to 56.3)

Data in parentheses are 95% Uncertainty Intervals (95% UIs)

Supplementary table 4

Country	Measure	Attributed age-standardized rate (per 100,000)						% Change (1990 to 2019)		
		1990			2019					
		Both	Female	Male	Both	Female	Male	Both	Female	Male
Afghanistan	Deaths	10.02 (5.08 to 17.64)	2.38 (1.55 to 4.23)	16.83 (7.88 to 30.58)	9.42 (5.89 to 15.19)	3.09 (2.1 to 4.41)	16.5 (9.71 to 28.34)	-6 (-33.3 to 42.1)	29.9 (-21.7 to 114.2)	-1.9 (-31 to 56.2)
	DALYs	238.15 (110.44 to 427.09)	62.12 (40.13 to 114.05)	399.49 (168.62 to 749.97)	223.27 (129.02 to 364.63)	79.41 (48.49 to 117.09)	385.06 (215.2 to 672.62)	-6.2 (-34.7 to 46.3)	27.8 (-23.4 to 110.4)	-3.6 (-34.5 to 59.1)
	YLLs	236.05 (109.5 to 423.76)	61.55 (39.74 to 113.13)	396.01 (166.83 to 743.13)	221.32 (127.72 to 361.48)	78.7 (47.98 to 116.07)	381.72 (213.39 to 666.94)	-6.2 (-34.8 to 46.1)	27.9 (-23.4 to 110.5)	-3.6 (-34.5 to 59.2)
	YLDs	2.1 (0.89 to 4)	0.57 (0.32 to 1.05)	3.48 (1.31 to 6.93)	1.95 (1.04 to 3.32)	0.71 (0.4 to 1.15)	3.34 (1.65 to 6.14)	-7.1 (-37.3 to 52.1)	25 (-26 to 107.7)	-3.9 (-37.6 to 73.5)
Algeria	Deaths	10.42 (8.4 to 12.93)	2.14 (1.63 to 2.79)	18.4 (14.8 to 23.12)	8.35 (6.42 to 10.78)	2.36 (1.75 to 3.13)	13.93 (10.53 to 18.45)	-19.9 (-42.4 to 9.8)	9.8 (-21.1 to 46.9)	-24.3 (-46.7 to 6.9)
	DALYs	227.54 (180.81 to 286.55)	48.08 (35.76 to 62.68)	410.4 (323.6 to 521.56)	180.16 (135.65 to 235.81)	50.1 (36.54 to 67.14)	304.77 (226.69 to 407.43)	-20.8 (-44.4 to 10.9)	4.2 (-24.9 to 41.9)	-25.7 (-48.8 to 6.4)
	YLLs	225.39 (179.26 to 284.35)	47.61 (35.42 to 62.04)	406.58 (320.44 to 514.96)	178.4 (134.49 to 234.06)	49.57 (36.16 to 66.49)	301.85 (224.5 to 403.53)	-20.8 (-44.4 to 11)	4.1 (-24.9 to 41.7)	-25.8 (-48.7 to 6.4)
	YLDs	2.15 (1.35 to 3.03)	0.47 (0.29 to 0.69)	3.82 (2.41 to 5.45)	1.76 (1.12 to 2.57)	0.54 (0.34 to 0.79)	2.92 (1.78 to 4.38)	-18.1 (-44.7 to 20.5)	14 (-24.1 to 63.8)	-23.6 (-50.2 to 17.1)
Bahrain	Deaths	39.69 (33.44 to 46.02)	13.03 (10.7 to 15.92)	66.59 (55.44 to 77.74)	18.6 (14.15 to 23.8)	8.59 (6.54 to 11.1)	27.79 (20.81 to 36.24)	-53.1 (-66.1 to -35.3)	-34.1 (-49.9 to -12.3)	-58.3 (-70.6 to -40.1)
	DALYs	772.75 (650.71 to 901.45)	254.89 (209.36 to 308.36)	1248.58 (1043.44 to 1464.19)	325.39 (246.87 to 423.26)	153.59 (116.54 to 197.72)	465.29 (346.63 to 618.01)	-57.9 (-69.8 to -40.5)	-39.7 (-55.3 to -19.4)	-62.7 (-74 to -45.9)
	YLLs	765.35 (644.47 to 892.37)	252.41 (207.59 to 304.84)	1236.48 (1031.92 to 1447.78)	321.94 (243.99 to 418.78)	151.83 (115.26 to 195.01)	460.37 (343.16 to 611.59)	-57.9 (-69.9 to -40.5)	-39.8 (-55.4 to -19.8)	-62.8 (-74 to -45.9)
	YLDs	7.39 (5.08 to 9.73)	2.48 (1.63 to 3.51)	12.1 (8.25 to 15.99)	3.45 (2.27 to 4.88)	1.76 (1.14 to 2.56)	4.92 (3.18 to 7.1)	-53.3 (-67.7 to -32.3)	-29 (-52.7 to 2.7)	-59.4 (-72.8 to -39.6)

Country	Measure	Attributed age-standardized rate (per 100,000)						% Change (1990 to 2019)		
		1990			2019					
		Both	Female	Male	Both	Female	Male	Both	Female	Male
Egypt	Deaths	5.1 (4.51 to 5.66)	1.81 (1.49 to 2.14)	8.38 (7.33 to 9.36)	7.24 (5.14 to 9.86)	3.32 (2 to 5.09)	10.49 (7.25 to 14.63)	42.1 (-3.1 to 95.2)	83.7 (12.3 to 168.4)	25.2 (-16.4 to 77.4)
	DALYs	130.21 (115.54 to 145.14)	44.61 (36.9 to 52.44)	214.97 (188.55 to 241.64)	179.85 (126.76 to 245.78)	77.28 (47.52 to 117.07)	268.73 (182.29 to 379.26)	38.1 (-6.6 to 89.9)	73.2 (7.5 to 151.2)	25 (-16.9 to 77.8)
	YLLs	129.06 (114.78 to 143.99)	44.18 (36.53 to 51.93)	213.09 (187.4 to 239.14)	178.22 (125.45 to 244.25)	76.54 (47.14 to 115.78)	266.35 (181.02 to 375.76)	38.1 (-6.6 to 89.8)	73.2 (7.6 to 151.4)	25 (-17.1 to 77.9)
	YLDs	1.16 (0.75 to 1.59)	0.43 (0.29 to 0.59)	1.88 (1.21 to 2.66)	1.63 (0.94 to 2.53)	0.75 (0.39 to 1.21)	2.38 (1.31 to 3.82)	40.7 (-13.1 to 111.1)	73.5 (0.5 to 164.8)	26.5 (-25.1 to 100.7)
Iran (Islamic Republic of)	Deaths	9.01 (7.32 to 11.02)	2.51 (1.98 to 3.42)	15.42 (12.33 to 18.87)	9.36 (8.44 to 10.31)	4.12 (3.35 to 4.98)	14.62 (13.29 to 16.08)	3.9 (-19.6 to 34.8)	64.3 (8.8 to 109.1)	-5.2 (-26.9 to 26.4)
	DALYs	206.73 (168.64 to 251.79)	58.31 (46.56 to 79.32)	344.92 (278.41 to 421.9)	204.58 (185.27 to 224.75)	89.59 (74.19 to 106.8)	321.01 (290.82 to 352.98)	-1 (-23.3 to 27.5)	53.6 (0.9 to 93.3)	-6.9 (-28.8 to 23.4)
	YLLs	204.83 (166.92 to 249.25)	57.75 (46.24 to 78.51)	341.76 (275.47 to 417.74)	202.59 (183.54 to 222.86)	88.64 (73.25 to 105.73)	317.97 (287.88 to 349.68)	-1.1 (-23.3 to 27.4)	53.5 (0.8 to 93.2)	-7 (-28.9 to 23.4)
	YLDs	1.89 (1.28 to 2.62)	0.56 (0.37 to 0.83)	3.17 (2.12 to 4.4)	1.99 (1.41 to 2.61)	0.95 (0.65 to 1.3)	3.04 (2.15 to 3.98)	5.1 (-18.3 to 34.7)	70.1 (10.1 to 116.2)	-4 (-26.7 to 27.2)
Iraq	Deaths	14.61 (11.69 to 18.26)	3.25 (2.22 to 5.41)	26.78 (20.73 to 33.7)	16.54 (12.96 to 20.03)	6.1 (4.53 to 8)	28.07 (21.97 to 33.54)	13.2 (-15.3 to 50.4)	87.7 (2.2 to 197)	4.8 (-22 to 40.3)
	DALYs	345.08 (271.9 to 431.19)	80.61 (55.69 to 135.7)	620.25 (473.75 to 787.63)	367.6 (280.43 to 457.6)	142.23 (103.81 to 189.57)	605.01 (461.12 to 738.92)	6.5 (-23 to 45.6)	76.5 (-5.6 to 182.7)	-2.5 (-30.3 to 33.8)
	YLLs	342.03 (269.04 to 427.22)	79.86 (55.16 to 134.38)	614.77 (470.25 to 781.41)	364.18 (278.28 to 453.73)	140.86 (102.8 to 187.79)	599.39 (456.79 to 733.11)	6.5 (-23 to 45.6)	76.4 (-5.7 to 182.3)	-2.5 (-30.4 to 33.8)
	YLDs	3.05 (1.98 to 4.29)	0.75 (0.43 to 1.26)	5.48 (3.49 to 7.88)	3.42 (2.22 to 4.91)	1.38 (0.86 to 2.13)	5.61 (3.63 to 8.1)	12 (-23.3 to 59.3)	83.4 (-4.1 to 223.1)	2.5 (-31.7 to 49.8)

Country	Measure	Attributed age-standardized rate (per 100,000)						% Change (1990 to 2019)		
		1990			2019					
		Both	Female	Male	Both	Female	Male	Both	Female	Male
Jordan	Deaths	11.55 (9.31 to 14.3)	2.98 (2.27 to 4.16)	20.07 (15.97 to 24.95)	12.81 (10.36 to 15.68)	4.5 (3.46 to 5.89)	20.43 (15.96 to 25.49)	10.9 (-17.3 to 48.9)	51 (1.1 to 119.4)	1.8 (-26.9 to 41)
	DALYs	273.09 (221.09 to 339.08)	68.7 (52.4 to 94.19)	469.29 (372.39 to 589.49)	281.44 (226.98 to 344.87)	96.94 (74.18 to 127.31)	448.81 (344.41 to 566.81)	3.1 (-24.8 to 39.6)	41.1 (-5.1 to 104.5)	-4.4 (-32.6 to 35.1)
	YLLs	270.62 (218.74 to 336.23)	68.04 (51.9 to 93.34)	465.06 (368.91 to 584.34)	278.71 (224.47 to 341.9)	95.9 (73.43 to 125.89)	444.54 (341.61 to 561.55)	3 (-24.9 to 39.6)	41 (-5.2 to 104.2)	-4.4 (-32.8 to 35.1)
	YLDs	2.47 (1.69 to 3.45)	0.67 (0.42 to 1.01)	4.23 (2.83 to 5.93)	2.72 (1.78 to 3.82)	1.04 (0.66 to 1.6)	4.27 (2.67 to 6.12)	10.3 (-22.7 to 57.3)	55.8 (-1.6 to 142.3)	0.9 (-32.6 to 50.1)
Kuwait	Deaths	10.37 (9.18 to 11.81)	4.09 (3.1 to 5.4)	14.69 (12.95 to 17.02)	9.07 (7.21 to 11.13)	3 (2.28 to 3.89)	13.01 (10.06 to 16.16)	-12.5 (-29.2 to 8.8)	-26.5 (-47.8 to 2.2)	-11.4 (-29.6 to 12.5)
	DALYs	235.95 (209.39 to 266.29)	92.48 (70.95 to 121.37)	323.93 (285.12 to 372.98)	177.96 (142.41 to 217.02)	61.93 (47.43 to 80.29)	254.62 (196.66 to 320.22)	-24.6 (-38.9 to -5.8)	-33 (-53.2 to -4.8)	-21.4 (-38.4 to -0.2)
	YLLs	233.69 (207.1 to 263.65)	91.53 (70.24 to 119.91)	320.8 (282.04 to 369.32)	176.08 (140.6 to 214.87)	61.18 (46.87 to 79.33)	251.99 (194.92 to 317.15)	-24.7 (-39 to -6)	-33.2 (-53.2 to -4.8)	-21.4 (-38.4 to -0.4)
	YLDs	2.27 (1.54 to 3.12)	0.95 (0.58 to 1.41)	3.12 (2.08 to 4.31)	1.89 (1.24 to 2.66)	0.75 (0.44 to 1.15)	2.63 (1.69 to 3.78)	-16.8 (-39.9 to 13.7)	-20.6 (-51.8 to 26.5)	-15.8 (-42 to 20.4)
Lebanon	Deaths	18.65 (13.96 to 23.74)	6.17 (4.95 to 7.8)	31.6 (22.51 to 41.18)	23.89 (19.52 to 31.6)	13.09 (9.48 to 18.18)	37.08 (29.79 to 49.33)	28.1 (-8 to 97.1)	112.2 (27.5 to 196.6)	17.4 (-17.7 to 89.5)
	DALYs	440.5 (322.98 to 564.75)	146.27 (115.39 to 187.82)	737.09 (512.4 to 971.07)	537.4 (430.6 to 702.43)	295.72 (212.4 to 401.48)	830.28 (652.81 to 1095.85)	22 (-13.6 to 88.4)	102.2 (24.1 to 181.1)	12.6 (-22.3 to 84.1)
	YLLs	436.61 (319.9 to 560.07)	144.91 (114.54 to 186.16)	730.61 (508.27 to 962.17)	532.13 (426.17 to 695.95)	292.43 (210.25 to 396.98)	822.6 (647.47 to 1086.14)	21.9 (-13.7 to 88.5)	101.8 (23.8 to 180.2)	12.6 (-22.3 to 84.2)
	YLDs	3.9 (2.44 to 5.62)	1.36 (0.86 to 2.04)	6.48 (3.84 to 9.68)	5.27 (3.54 to 7.56)	3.29 (2.05 to 4.9)	7.68 (5.05 to 11.39)	35.2 (-9.2 to 111.1)	141.4 (38.4 to 275.7)	18.5 (-24.1 to 98)

Country	Measure	Attributed age-standardized rate (per 100,000)						% Change (1990 to 2019)		
		1990			2019					
		Both	Female	Male	Both	Female	Male	Both	Female	Male
Libya	Deaths	18.09 (13.71 to 23.13)	1.5 (1.02 to 2.44)	33.16 (25.02 to 42.68)	16.24 (11.96 to 20.89)	2.51 (1.52 to 3.64)	29.54 (21.64 to 37.91)	-10.2 (-38.8 to 31.3)	67.3 (-31.2 to 176.1)	-10.9 (-39.6 to 31.2)
	DALYs	430.16 (325.67 to 553.98)	36.12 (24.76 to 59.5)	774.33 (585.92 to 1006.66)	378.6 (277.38 to 488.27)	59.84 (36.13 to 87.52)	685.25 (498.72 to 887.26)	-12 (-40.4 to 30.4)	65.7 (-31.5 to 176.3)	-11.5 (-40.7 to 33)
	YLLs	426.35 (322.75 to 548.95)	35.75 (24.49 to 58.99)	767.45 (580.61 to 997.64)	375.2 (274.74 to 483.97)	59.25 (35.83 to 86.73)	679.12 (494.28 to 878.78)	-12 (-40.4 to 30.5)	65.7 (-31.4 to 176.2)	-11.5 (-40.7 to 32.9)
	YLDs	3.81 (2.49 to 5.42)	0.36 (0.22 to 0.62)	6.88 (4.46 to 9.82)	3.4 (2.16 to 4.88)	0.59 (0.31 to 0.95)	6.12 (3.85 to 8.91)	-10.7 (-43 to 32.8)	61.2 (-35.1 to 179)	-11 (-44.8 to 33.9)
Morocco	Deaths	12.11 (9.09 to 14.95)	1.22 (0.89 to 1.67)	23.27 (17.33 to 29.06)	13.07 (9.35 to 16.86)	1.98 (1.31 to 2.73)	24.36 (17.18 to 31.6)	7.9 (-22.8 to 45.7)	61.8 (5.8 to 133.5)	4.7 (-25.8 to 43.3)
	DALYs	308.77 (234.42 to 380.77)	29.23 (21.68 to 39.34)	593.16 (447.16 to 737.22)	321.87 (228.15 to 419.7)	46.06 (30.99 to 65.24)	599.23 (423.77 to 791.59)	4.2 (-27.7 to 44.1)	57.6 (0.6 to 131.8)	1 (-29.9 to 40.8)
	YLLs	306.05 (232.9 to 377.72)	28.94 (21.47 to 38.98)	587.95 (443.34 to 731.48)	319.02 (226.14 to 416.27)	45.61 (30.74 to 64.67)	593.97 (420.02 to 785.09)	4.2 (-27.6 to 44)	57.6 (0.5 to 131.8)	1 (-29.8 to 40.9)
	YLDs	2.72 (1.75 to 3.83)	0.29 (0.18 to 0.43)	5.2 (3.29 to 7.35)	2.84 (1.77 to 4.19)	0.46 (0.28 to 0.7)	5.25 (3.19 to 7.9)	4.5 (-31 to 52.1)	59.5 (1.4 to 133.9)	1 (-34.4 to 50)
Oman	Deaths	7.72 (5.66 to 9.8)	1.99 (1.27 to 3.26)	13.67 (10.01 to 17.34)	7.5 (6 to 9.46)	3.17 (2.24 to 4.13)	11.63 (9.2 to 14.86)	-2.8 (-30.5 to 40.4)	59.1 (-23.9 to 165.9)	-14.9 (-40 to 26.7)
	DALYs	178.19 (129.11 to 229.76)	45.35 (28.49 to 74.66)	297.42 (215.12 to 384.37)	149.46 (116.86 to 196.25)	66.2 (46.87 to 86.34)	221.61 (168.89 to 299.72)	-16.1 (-41.9 to 24.6)	46 (-30.5 to 135)	-25.5 (-49.1 to 15)
	YLLs	176.54 (127.85 to 227.45)	44.89 (28.25 to 73.76)	294.63 (213.23 to 380.93)	147.9 (115.73 to 194.41)	65.45 (46.5 to 85.3)	219.33 (166.74 to 296.67)	-16.2 (-42 to 24.5)	45.8 (-30.6 to 134.9)	-25.6 (-49.1 to 14.9)
	YLDs	1.65 (1.02 to 2.49)	0.46 (0.24 to 0.78)	2.79 (1.72 to 4.21)	1.55 (1.01 to 2.26)	0.75 (0.44 to 1.14)	2.28 (1.44 to 3.4)	-5.8 (-37.8 to 47.1)	64.6 (-23.5 to 187.6)	-18.3 (-48.8 to 35.2)

Country	Measure	Attributed age-standardized rate (per 100,000)						% Change (1990 to 2019)		
		1990			2019					
		Both	Female	Male	Both	Female	Male	Both	Female	Male
Palestine	Deaths	18.18 (13.8 to 23.82)	2.94 (1.93 to 4.66)	36.75 (27.9 to 48.05)	19.46 (16.61 to 22.8)	4.96 (3.78 to 6.42)	36.73 (31.42 to 42.84)	7 (-23.3 to 48.3)	69 (-0.6 to 174.1)	-0.1 (-28.1 to 38.3)
	DALYs	412.17 (306 to 544.33)	71.03 (46.8 to 114.61)	826.06 (612.03 to 1098.1)	437.78 (371.78 to 515.86)	112.52 (85.21 to 144.97)	791.17 (671.26 to 933.74)	6.2 (-26.1 to 48.8)	58.4 (-6.6 to 160.5)	-4.2 (-32.9 to 34.1)
	YLLs	408.47 (303.4 to 540.06)	70.36 (46.45 to 113.77)	818.66 (605.12 to 1089.26)	433.8 (368.43 to 511.24)	111.41 (84.29 to 143.61)	783.93 (665.98 to 924.73)	6.2 (-26.2 to 48.9)	58.3 (-6.7 to 160.4)	-4.2 (-32.8 to 33.9)
	YLDs	3.71 (2.36 to 5.46)	0.67 (0.36 to 1.16)	7.39 (4.62 to 10.93)	3.99 (2.74 to 5.45)	1.12 (0.72 to 1.64)	7.24 (4.91 to 9.86)	7.6 (-26.4 to 59.5)	67.1 (-7.5 to 191.9)	-2.1 (-34.7 to 46.5)
Qatar	Deaths	15.8 (11.87 to 20.83)	3.47 (2.23 to 5.3)	25.72 (18.42 to 33.82)	17.15 (13.22 to 21.71)	6.87 (4.9 to 9.33)	20.63 (15.69 to 26.35)	8.5 (-28.1 to 62.3)	97.8 (15.8 to 222.2)	-19.8 (-47 to 26.9)
	DALYs	340.8 (251.21 to 452.84)	75.77 (50.95 to 112.25)	514.78 (369.09 to 685.03)	308.09 (228.06 to 408.67)	127.16 (90.55 to 173.73)	370.16 (271.5 to 499.3)	-9.6 (-43.5 to 43.6)	67.8 (3.8 to 157.3)	-28.1 (-55.7 to 20.3)
	YLLs	337.63 (248.54 to 448.88)	75.04 (50.48 to 111.42)	509.87 (365.26 to 679.09)	304.84 (224.59 to 405.48)	125.63 (89.49 to 171.31)	366.33 (268.88 to 494.26)	-9.7 (-43.7 to 43.5)	67.4 (3.6 to 156.8)	-28.2 (-55.7 to 20.3)
	YLDs	3.17 (2 to 4.5)	0.74 (0.4 to 1.23)	4.92 (3.05 to 7.07)	3.25 (2.12 to 4.76)	1.53 (0.93 to 2.31)	3.84 (2.51 to 5.7)	2.4 (-37.5 to 65)	106.8 (14.3 to 250.9)	-22 (-53.5 to 33.7)
Saudi Arabia	Deaths	6.81 (4.95 to 8.86)	1.79 (1.16 to 3.08)	10.65 (7.71 to 13.89)	7.08 (5.52 to 8.75)	2.92 (2.1 to 3.89)	9.84 (7.62 to 12.31)	4.1 (-29.3 to 56.1)	63 (-17.7 to 174.9)	-7.6 (-37.7 to 43.1)
	DALYs	157.17 (113.29 to 209.25)	43.33 (28.2 to 71.94)	238.37 (168.79 to 316.2)	159.45 (121.92 to 197.32)	67.96 (48.72 to 90.94)	218.92 (165.1 to 274.06)	1.5 (-32.2 to 53.8)	56.9 (-19.5 to 165.9)	-8.2 (-39.5 to 44.5)
	YLLs	155.84 (112.23 to 207.63)	42.97 (27.96 to 71.08)	236.33 (167.79 to 313.45)	157.9 (120.45 to 195.28)	67.24 (48.16 to 89.84)	216.83 (163.45 to 271.4)	1.3 (-32.4 to 53.4)	56.5 (-19.7 to 165.2)	-8.2 (-39.6 to 44.5)
	YLDs	1.33 (0.83 to 2)	0.36 (0.21 to 0.65)	2.04 (1.24 to 3.04)	1.55 (0.99 to 2.27)	0.72 (0.44 to 1.08)	2.09 (1.28 to 3.16)	16.3 (-23.2 to 88)	99 (1.1 to 236)	2.2 (-37.3 to 76.4)

Country	Measure	Attributed age-standardized rate (per 100,000)						% Change (1990 to 2019)		
		1990			2019					
		Both	Female	Male	Both	Female	Male	Both	Female	Male
Sudan	Deaths	6.82 (3.9 to 12.59)	1.63 (1.03 to 2.53)	11.55 (5.9 to 22.1)	6.95 (4.54 to 11.27)	2.57 (1.8 to 3.47)	10.55 (6.51 to 18.15)	1.8 (-30.7 to 58.7)	58.2 (-8.6 to 164.1)	-8.6 (-39.3 to 48.1)
	DALYs	160.57 (87.76 to 298.81)	40.23 (26.62 to 60.01)	268.51 (131.51 to 521)	157.88 (98.21 to 256.49)	61.54 (41.26 to 86.36)	238.02 (141.92 to 412.23)	-1.7 (-34.3 to 62.3)	53 (-12.4 to 156.9)	-11.4 (-42.7 to 51.3)
	YLLs	159.1 (87.2 to 295.65)	39.84 (26.38 to 59.34)	266.06 (130.08 to 516)	156.38 (97.28 to 253.61)	60.94 (40.84 to 85.37)	235.78 (140.53 to 407.48)	-1.7 (-34.3 to 62.4)	53 (-12.5 to 156.9)	-11.4 (-42.8 to 51.3)
	YLDs	1.47 (0.68 to 3)	0.39 (0.21 to 0.64)	2.45 (1 to 5.3)	1.5 (0.85 to 2.64)	0.6 (0.35 to 0.91)	2.25 (1.2 to 4.21)	1.8 (-34.9 to 73.5)	55.7 (-15 to 163.8)	-8.5 (-43.9 to 67.5)
Syrian Arab Republic	Deaths	8.66 (6.62 to 10.85)	2.49 (1.77 to 3.54)	14.2 (10.83 to 17.98)	9.07 (6.73 to 11.87)	3.58 (2.43 to 4.89)	14.12 (10.38 to 18.62)	4.7 (-28 to 52)	43.8 (-22.5 to 132)	-0.6 (-31.9 to 47.7)
	DALYs	208.34 (157.12 to 263.06)	61.08 (43.47 to 84.33)	342.2 (255.14 to 439.54)	208.08 (152.4 to 280.44)	78.45 (52.5 to 110.47)	332.05 (240.67 to 444.74)	-0.1 (-32.3 to 48.4)	28.4 (-31.2 to 106.2)	-3 (-34.7 to 47.7)
	YLLs	206.45 (155.95 to 260.57)	60.51 (43.03 to 83.33)	339.11 (252.8 to 434.81)	206.12 (151.06 to 277.21)	77.64 (52.04 to 109.35)	328.99 (239.03 to 440.63)	-0.2 (-32.3 to 48.2)	28.3 (-31.2 to 106)	-3 (-34.7 to 47.9)
	YLDs	1.89 (1.19 to 2.73)	0.57 (0.35 to 0.88)	3.09 (1.9 to 4.48)	1.97 (1.23 to 2.91)	0.81 (0.47 to 1.22)	3.06 (1.85 to 4.61)	4 (-30.7 to 63.3)	41.8 (-25.3 to 140.1)	-0.9 (-36.5 to 56.7)
Tunisia	Deaths	17.95 (14.35 to 21.72)	1.39 (1.03 to 1.84)	33.64 (26.97 to 40.7)	17.36 (12.25 to 23.93)	2.07 (1.46 to 2.89)	33.77 (23.77 to 46.51)	-3.3 (-35.5 to 44.3)	49.1 (-4.1 to 125.6)	0.4 (-33.7 to 50.4)
	DALYs	407.83 (325.91 to 493.27)	32.46 (24.34 to 43.12)	759.98 (604.72 to 922.44)	400.78 (279.46 to 558.54)	46.81 (33.15 to 65.99)	769.7 (530.52 to 1074.67)	-1.7 (-36 to 46.7)	44.2 (-5.6 to 119)	1.3 (-34.2 to 51.6)
	YLLs	404.12 (323.18 to 488.82)	32.13 (24.12 to 42.67)	753.09 (599.85 to 914.16)	397.1 (276.24 to 553.38)	46.29 (32.78 to 65.14)	762.69 (524.42 to 1063.6)	-1.7 (-36 to 46.6)	44.1 (-5.7 to 118.7)	1.3 (-34.3 to 51.6)
	YLDs	3.72 (2.43 to 5.19)	0.33 (0.21 to 0.5)	6.89 (4.48 to 9.65)	3.68 (2.16 to 5.59)	0.52 (0.31 to 0.83)	7 (4.1 to 10.73)	-1.1 (-36.6 to 53.5)	55.9 (0.4 to 142.5)	1.6 (-35.7 to 59.7)

Country	Measure	Attributed age-standardized rate (per 100,000)						% Change (1990 to 2019)		
		1990			2019					
		Both	Female	Male	Both	Female	Male	Both	Female	Male
Turkey	Deaths	35.06 (27.68 to 43.39)	5.64 (4.46 to 7.61)	67.54 (52.59 to 84.37)	28.99 (22.84 to 36)	6.87 (5.29 to 8.76)	54.17 (42.61 to 67.52)	-17.3 (-41.3 to 14)	21.7 (-15.5 to 71.3)	-19.8 (-44 to 12.2)
	DALYs	895.44 (690.6 to 1115.37)	141.13 (112.05 to 191.92)	1701.36 (1296.64 to 2149.78)	697.24 (547.57 to 872.4)	158.39 (121.66 to 203.55)	1288.33 (1008.8 to 1622.99)	-22.1 (-45.5 to 9.2)	12.2 (-22.8 to 56.8)	-24.3 (-48 to 8.6)
	YLLs	887.85 (684.98 to 1106.71)	139.86 (111.3 to 190.02)	1686.93 (1287.13 to 2131.72)	690.88 (542.12 to 863.7)	156.72 (120.38 to 201.5)	1276.73 (1000.35 to 1608.1)	-22.2 (-45.6 to 9)	12.1 (-22.9 to 56.5)	-24.3 (-48 to 8.5)
	YLDs	7.58 (4.98 to 10.66)	1.27 (0.8 to 1.91)	14.43 (9.35 to 20.56)	6.36 (4.31 to 9.02)	1.68 (1.07 to 2.52)	11.59 (7.83 to 16.48)	-16.1 (-42.7 to 20.5)	31.8 (-14.2 to 98.5)	-19.7 (-46.1 to 16.8)
United Arab Emirates	Deaths	16.41 (11.99 to 21.12)	6.24 (3.49 to 11.26)	23.81 (17.06 to 30.35)	15.91 (11.47 to 22.27)	8.04 (4.37 to 13.05)	19.62 (14.31 to 27.4)	-3.1 (-35.1 to 53.7)	28.8 (-53.3 to 99.5)	-17.6 (-45.6 to 40)
	DALYs	332.56 (245.08 to 430.16)	126.88 (73.2 to 221.06)	474.03 (339.18 to 614.49)	316.57 (227.82 to 440.51)	162.36 (94.46 to 251.4)	385.21 (279.72 to 536.15)	-4.8 (-37.3 to 50.2)	28 (-52 to 99.5)	-18.7 (-47.4 to 37.9)
	YLLs	329.38 (243.02 to 426.45)	125.64 (72.4 to 219.19)	469.48 (336.04 to 608.5)	313.49 (225.92 to 437.24)	160.72 (93.62 to 248.83)	381.46 (276.97 to 531.09)	-4.8 (-37.3 to 50.2)	27.9 (-52 to 98.9)	-18.7 (-47.4 to 37.8)
	YLDs	3.18 (1.97 to 4.59)	1.24 (0.58 to 2.39)	4.55 (2.77 to 6.51)	3.09 (1.88 to 4.83)	1.64 (0.81 to 2.87)	3.75 (2.26 to 5.77)	-3 (-40 to 58.4)	32.6 (-48.8 to 128.9)	-17.7 (-49.7 to 45.8)
Yemen	Deaths	8.56 (5.49 to 13.59)	1.85 (1.08 to 3.29)	16.75 (10.24 to 26.87)	8.89 (6.06 to 13.29)	2.84 (2.08 to 3.87)	15.18 (9.95 to 23.63)	3.8 (-28.3 to 58.6)	53.6 (-20.5 to 170.8)	-9.3 (-38.7 to 39.7)
	DALYs	210.2 (130.37 to 336.68)	44.93 (27.23 to 79.6)	389.15 (232.81 to 637.73)	205.33 (140.04 to 308.77)	68.25 (49.97 to 93.41)	345.95 (227.56 to 545.2)	-2.3 (-34.1 to 52.6)	51.9 (-22.2 to 168.8)	-11.1 (-41.6 to 42.7)
	YLLs	208.33 (129.17 to 333.89)	44.49 (26.97 to 78.81)	385.63 (230.58 to 631.77)	203.42 (138.73 to 306.17)	67.59 (49.38 to 92.7)	342.76 (225.52 to 539.39)	-2.4 (-34.1 to 52.3)	51.9 (-22.1 to 168.9)	-11.1 (-41.4 to 42.7)
	YLDs	1.88 (1.01 to 3.25)	0.44 (0.22 to 0.81)	3.52 (1.79 to 6.29)	1.91 (1.11 to 3.12)	0.66 (0.42 to 0.98)	3.2 (1.77 to 5.47)	1.5 (-36.3 to 64.6)	50.5 (-22.9 to 170.5)	-9.3 (-45.5 to 54.4)

Data in parentheses are 95% Uncertainty Intervals (95% UIs)
