# Supplementary material for: Burden of tracheal, bronchus, and lung cancer in North Africa and Middle East countries, 1990 to 2019: Results from the GBD study 2019
Source: Front Oncol. 2023 Feb 10;12:1098218. doi: 10.3389/fonc.2022.1098218 (PMC9951096; doi:10.3389/fonc.2022.1098218)
Supplement: Supplementary file 2 [file Image_1.pdf]

Deaths ranking

A

Risk factor	North Africa and Middle East	Afghanistan	Algeria	Bahrain	Egypt	Iran (Islamic Republic of)	Iraq	Jordan	Kuwait	Lebanon	Libya	Morocco	Oman	Palestine	Qatar	Saudi Arabia	Sudan	Syrian Arab Republic	Tunisia	Turkey	United Arab Emirates	Yemen
**Smoking**	1	1	1	1	1	1	1	1	1	1	1	1	1	1	1	1	1	1	1	1	1	1
**Particulate matter pollution**	2	2	2	2	2	2	2	2	2	2	2	2	2	2	2	2	2	2	2	3	2	2
**Occupational carcinogens**	3	4	5	4	5	5	4	4	4	5	5	4	3	5	4	5	5	5	5	2	5	5
**High fasting plasma glucose**	4	3	3	3	3	3	3	3	3	3	3	3	4	3	3	3	3	3	3	4	3	3
**Secondhand smoke**	5	5	4	5	4	4	5	5	5	4	4	6	5	4	5	4	4	4	4	5	4	4
**Residential radon**	6	7	6	6	7	6	7	6	7	6	6	5	6	6	6	7	7	6	6	6	6	7
**Diet low in fruits**	7	6	7	7	6	7	6	7	6	7	7	7	7	7	7	6	6	7	7	7	7	6

DALYs ranking

B

Risk factor	North Africa and Middle East	Afghanistan	Algeria	Bahrain	Egypt	Iran (Islamic Republic of)	Iraq	Jordan	Kuwait	Lebanon	Libya	Morocco	Oman	Palestine	Qatar	Saudi Arabia	Sudan	Syrian Arab Republic	Tunisia	Turkey	United Arab Emirates	Yemen
**Smoking**	1	1	1	1	1	1	1	1	1	1	1	1	1	1	1	1	1	1	1	1	1	1
**Particulate matter pollution**	2	2	2	2	2	2	2	2	2	2	2	2	2	2	2	2	2	2	2	3	2	2
**Occupational carcinogens**	3	4	5	4	4	5	4	4	4	5	5	4	3	5	4	5	5	4	4	2	5	5
**High fasting plasma glucose**	4	3	3	3	3	3	3	3	3	3	3	3	4	3	3	3	3	3	3	4	3	4
**Secondhand smoke**	5	5	4	5	5	4	5	5	5	4	4	6	5	4	5	4	4	5	5	5	4	3
**Residential radon**	6	7	6	6	7	6	7	6	7	6	6	5	6	6	6	7	7	6	6	6	6	7
**Diet low in fruits**	7	6	7	7	6	7	6	7	6	7	7	7	7	7	7	6	6	7	7	7	7	6
